# Drivers of COVID-19 Outcomes in Long-Term Care Facilities Using Multi-Level Analysis: A Systematic Review

**DOI:** 10.3390/healthcare12070807

**Published:** 2024-04-08

**Authors:** Mehri Karimi-Dehkordi, Heather M. Hanson, James Silvius, Adrian Wagg

**Affiliations:** 1 Faculty of Medicine & Dentistry, Keyano College, University of Alberta, Edmonton, AB T6G 2R3, Canada; 2 Seniors Health Strategic Clinical Network, Alberta Health Services, Cumming School of Medicine, University of Calgary, Calgary, AB T2N 1N4, Canada; heather.hanson@ahs.ca (H.M.H.); James.Silvius@albertahealthservices.ca (J.S.); 3 Seniors Health Strategic Clinical Network, Alberta Health Services, Faculty of Medicine & Dentistry, University of Alberta, Edmonton, AB T6G 2R3, Canada; wagg@ualberta.ca

**Keywords:** COVID-19, outbreaks, mortality, outcomes, review, long-term care facilities

## Abstract

This study aimed to identify the individual, organizational, and environmental factors which contributed to COVID-19-related outcomes in long-term care facilities (LTCFs). A systematic review was conducted to summarize and synthesize empirical studies using a multi-level analysis approach to address the identified influential factors. Five databases were searched on 23 May 2023. To be included in the review, studies had to be published in peer-reviewed journals or as grey literature containing relevant statistical data. The Joanna Briggs Institute critical appraisal tool was employed to assess the methodological quality of each article included in this study. Of 2137 citations identified after exclusions, 99 records met the inclusion criteria. The predominant individual, organizational, and environmental factors that were most frequently found associated with the COVID-19 outbreak comprised older age, higher dependency level; lower staffing levels and lower star and subset domain ratings for the facility; and occupancy metrics and co-occurrences of outbreaks in counties and communities where the LTCFs were located, respectively. The primary individual, organizational, and environmental factors frequently linked to COVID-19-related deaths comprised age, and male sex; higher percentages of racial and ethnic minorities in LTCFs, as well as ownership types (including private, for-profit, and chain membership); and higher occupancy metrics and LTCF’s size and bed capacity, respectively. Unfolding the risk factors collectively may mitigate the risk of outbreaks and pandemic-related mortality in LTCFs during future endemic and pandemics through developing and improving interventions that address those significant factors.

## 1. Introduction

In unravelling the complexities of the impact of the COVID-19 pandemic on older adults in long-term care facilities (LTCFs), it becomes apparent that a comprehensive understanding of the substantial individual, organizational, and environmental factors is essential. The importance of this understanding is highlighted by the disproportionate impact and the huge toll of SARS-CoV-2 coronavirus (COVID-19) infection on older adults in LTCFs, especially those with underlying health conditions who experienced a range of predominantly adverse outcomes and death [1,2]. Thus, this study examined COVID-19 case(s)/outbreaks and COVID-related deaths among residents in LTCFs where the pandemic hit the hardest.

The COVID-19-related outcomes in LTCFs were highlighted in a systematic review, reporting an infection rate of 45% coupled with a 23% mortality rate [3]. Despite global efforts, by October 2021, the pandemic persisted and continued to spread; it appeared to differentially affect LTCFs worldwide, albeit with varying degrees of intensity. In the USA and across Europe, the proportion of COVID-19 cases per occupied LTCF bed ranged from 2.2% (in Finland) to 50% (in the USA) [4]. COVID-19-related mortality rates per total population were also widely variable, from 11% in the Czech Republic to 50% in Belgium [4]. Factors influencing COVID-19-related outcomes included variation in reporting information, the reliability, accuracy, and validity of the research, virus variants, healthcare infrastructure, population density, staffing levels and training, and geographic and cultural factors [5,6,7,8]. The phenomenon’s complexity was further evident as COVID-19 outcomes within a specific location fluctuated over time. For instance, the Canadian Institute for Health Information (CIHI) reported that in LTCFs, the count of COVID-19-related fatalities for March, April, May, and June 2020 were 13, 804, 3692, and 948, respectively [9].

Efforts have been made to dissect this intricate and complex phenomenon by either concentrating on a single domain or a particular topic within a domain separately or by examining all three domains/levels but failing to incorporate the relevant factors and synthesize them collectively comprehensively. In the existing body of literature, various drivers and predictors have been identified that can be classified as individual (e.g., age, sex), organizational (e.g., the ratio of staff to residents), and environmental factors (e.g., community prevalence). Reviews investigating determinants of COVID-19 outcomes in LTCFs have appeared in the scientific literature since 2020; to our knowledge, no published systematic review has yet collectively addressed these factors [10,11,12]. The synthesis of emerging research and a comprehensive appreciation of the individual and contextual factors which might influence COVID-19 outcomes is vital for effective management and mitigation of harm in future infectious outbreaks.

The aim of this systematic review was to identify the individual, organizational, and environmental factors which together might underly COVID-19-related outcomes in LTCF.

To achieve this goal, the following are research questions:

1. What individual factors or characteristics, such as Frailty Index and age, impacted COVID-19 outcomes within LTCFs?
2. What organizational characteristics and practices (e.g., staffing level, ownership status) within LTCFs affected the spread and mortality-related outcomes?
3. Did environmental factors, such as facility/room design and facility age, influence COVID-19 outbreak and death?

This approach prevents the skewing of the impact of one group of factors without considering their broader context and will suggest comprehensive areas that require further research.

## 2. Materials and Methods

The Preferred Reporting Items for Systematic reviews and Meta-Analyses statements (PRISMA-S) checklist guided the conduct and reporting of the current systematic review [13].

PROSPERO registration number: CRD42024519707.

### 2.1. Search Strategy and Data Sources

The systematic search was conducted with the collaboration of a librarian scientist using OVID MEDLINE, EMBASE, EBSCOhost CINAHL, and the Wiley Cochrane Database of Systematic Reviews. The search results included those published from inception to 23 May 2023. The details of search terms and strategies are shown in Table S1.

### 2.2. Eligibility Criteria

The PICO model was employed to design this review [14]; P (population): older adults residing in LTCFs or nursing homes regardless of sex and their health status, I (Intervention/exposure): LTCFs or nursing homes that reported case(s) with confirmed COVID-19-related outcomes, C (comparison): characteristics between LTCFs or residents with and without COVID-19-related outcomes, O (outcomes): the determinants that were associated with at least one confirmed COVID-19 case or death-related COVID-19 among residents in LTCFs.

To be included in the review, studies had to be original observational studies, published in peer-reviewed journals or as grey literature since 2020, and contain original statistical data on factors associated with COVID-19 outbreaks or COVID-19-related deaths among LTC residents. Studies were excluded if members of the study population were transferred to acute hospitals due to variations in care levels, staffing, and in the environment [15]. Inclusion of hospital transfers might introduce a potential sampling bias, for example, a study of hospitalized residents diagnosed with COVID-19 exhibited a significantly elevated mortality rate (ranging from 51.6% to 59.3%), contrasting with control samples (8.1% to 9.7%) within LTCFs [16]. Additional grounds for exclusion were met when the study’s participants resided in alternative types of facilities, such as retirement homes, where residents had limited medical and daily assistance needs, or when the studies were oriented towards assessing the effects of interventions on outcomes related to COVID-19. A search for publications in languages other than English confirmed that no substantial (>5%) body of literature was excluded from the review.

### 2.3. Data Extraction

After removing the duplicates and screening the titles and abstracts, the full text of all potentially eligible articles was retrieved and assessed against the inclusion criteria. The Joanna Briggs Institute (JBI) critical appraisal tools for cross-sectional studies (eight-item scale), case–control (ten-item scale), and cohort studies (eleven-item scale) were used to appraise the methodological quality of the included studies [17]. The studies were categorized into three levels based on quality assessment scores with scores ranging from zero to 8, 10, and 11 for each study type, respectively. Those in level one predominantly fulfilled the criteria and exhibited minimal bias risk, whereas level three studies failed to meet multiple criteria or demonstrated a significant risk of bias (Table S2). We also used a modified version of the JBI data extraction tool to include specific data about participants, context, methods, and outcomes (including individual, organizational, and environmental factors that may influence COVID-19 outbreaks and deaths). Prior to the study, the extraction tool was pilot tested on five studies by MKD and HMH using various methodological designs to make sure all related data were obtained. The tool was then refined and finalized through author consensus on conceptualizing and adding richness to the concepts of organizational, environmental, and individual factors using ecological models, focused on unravelling the complex interplay between factors which might influencing behaviour or care [18]. Each stage of the data collection was accompanied by team review and discussion until a consensus was reached.

### 2.4. Data Analysis

The multi-level analysis was employed to identify three levels of determinants by incorporating both deductive and inductive analysis [19]. The first author initially extracted tentative codes and themes. Then, the last author reviewed the results from each round and updated these. The analytical process was reviewed by the whole research team, and disagreements were resolved by discussion. We began by deductively coding the quantitative data against the three a priori codes: individual, organizational, and environmental factors. The data under each level were then categorized under two outcome groups: COVID-19 outbreaks and COVID-19-related deaths. Data under each category were then coded again, which led to emerging subcodes (the second level of the coding scheme) which revealed corresponding determinants for each level. Subcodes were then inductively clustered into two major categories for individual determinants and environmental factors and three major categories for organizational factors. The three a priori levels comprised seven themes and 98 subcodes. We synthesized data narratively and presented these under the three levels.

## 3. Results

**Figure 1:**
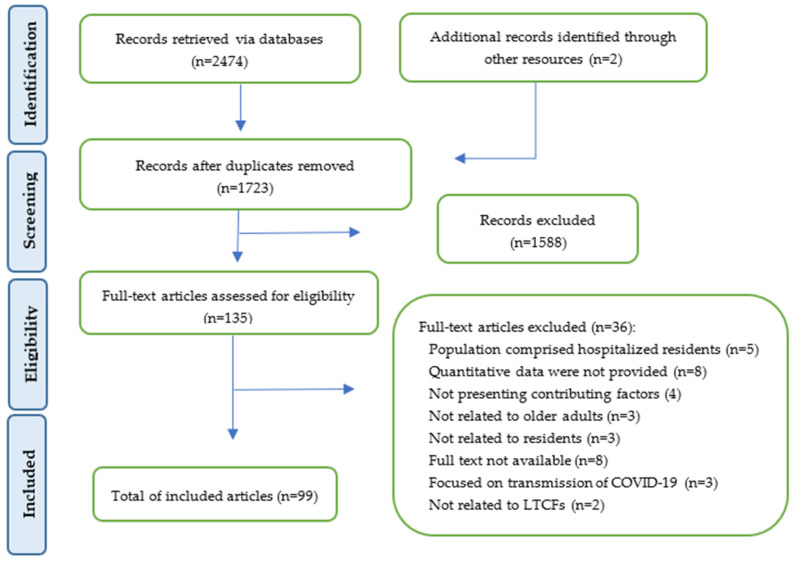
PRISMA flow-chart for the systematic review process.

Of the 2474 studies’ records identified via the systematic search strategy, 99 records met the inclusion criteria (Figure 1).

Data relating to the characteristics of the included studies are presented in Table 1.

Geographically, the studies originated from the USA (51), Canada (9), Spain (12), Italy (5), France (6), England (2), Australia (1), Ireland (1), Belgium (1), Netherlands (4), Andorra (1), Brazil (1), Germany (1), Korea (1), Switzerland (1), South Africa (1), and Wales (1). These studies followed an observational design and provided data on one or more of the strata of interest. Specifically, 45% of the articles focused on factors influencing both the occurrence of outbreaks and mortality; in comparison, 30% and 23% of the studies delved into the specifics of outbreaks and mortality outcomes, respectively.

A significant portion (40%) of the articles explored the impact of individual, organizational, and environmental factors concurrently.

The included articles examined these factors using 3 to 150 subcategorized variables, with a median of 23. Seventy-one percent of the articles were of moderate quality. Variability in findings was observed among studies classified as either low quality (6%) or high quality (22%). Variability in results was observed explicitly across articles that performed adjustment analyses or effect analyses (83%) and those that did not undertake them (16%).

The findings are organized into three primary sections, focusing on individual, organizational, and environmental factors. Within these sections, various subgroups are linked to both COVID-19 outbreak and COVID-related deaths. The presentation of data in Tables S3–S8 illustrates this structure, wherein each section is further divided into subsections emerged from an inductive analysis of the data.

### 3.1. Individual Factors

Individual factors were conceptualized as the attributes of LTCF residents that were related to COVID-19 infections and deaths.

#### 3.1.1. Individual Factors Related to Outbreaks

Identified individual factors were linked to 13 areas and fell into two groups: (1) sociodemographic background (age, sex, and socioeconomic status) and (2) condition-specific factors (comorbid conditions, scores of health status instruments, smokers, co-existent medications, seroprevalence, body mass index (BMI), resident dependency level, frailty index, duration of stay in LTCFs, and hospitalization experience).

The individual factors that were most supported by the body of evidence with relation to COVID-19 outbreak include age [24,39,46,60,63,97,101,105,115], dependency level [22,46,54,58,63,72,101,105], frailty [24,58,115], sex (female) [45,56,91], and cognitive deterioration/dementia [105,107,115].

Table S3 details the individual factors associated with outbreaks in LTCFs.

#### 3.1.2. Individual Factors Related to COVID-19-Related Deaths

Fifteen areas of individual factors associated with COVID-19-related death emerged and were categorized as follows: (1) sociodemographic background (age, sex, and social engagement level) and (2) condition-specific factors (cognitive/mental status, comorbidity, symptoms, lab test results, nutritional status, BMI, degree of dependence and level of needed care, frailty, co-existent medications, duration of stay in LTCFs, and hospitalization experience).

Several factors frequently linked to mortality include male sex [20,24,29,32,46,51,52,54,55,58,59,60,62,68,98,101,103], age [24,29,32,33,46,51,53,55,58,59,60,66,68,75,98,101,102,103,115], dependency and level of care needed, [2,20,32,44,46,54,56,63,101,103,110,113,116], cognitive deterioration [20,29,32,33,55,59,86,98,101,115], frailty [23,24,44,45,46,105,115,116], anticoagulation, antiplatelets, or antiplatelets and anticoagulants (inverse association) [20,29,32,46,52,68,98], comorbidity [24,29,52,55,58,98], cardiovascular disease [32,55,60,68,101], respiratory disease [45,59,60,101] hospitalization [29,58,98,113], chronic kidney disease [68,101,103,107], fever symptom [20,58,59,103], hypoxia/respiratory insufficiency symptoms [45,49,58,59,103], diabetes [101,103,107], hypertension [60,81,110], and immunocompromised status [20,29,98].

Table S4 details the individual factors associated with death in LTCFs.

### 3.2. Organizational Factors

Organizational factors were conceptualized as the internal attributes and organizational characteristics of an LTCF.

#### 3.2.1. Organizational Factors Related to Infection Outbreaks

Eight areas were identified across three groups: (1) LTCFs quality indicators (quality rating star, quality performance), (2) staffing (staffing levels, infected staff, nursing staff assignment, and employment status, (3) ownership and membership affiliation (types of ownership, chain membership), (4) Medicare and Medicaid coverage, and (5) LTCF’s racial and ethnic composition.

The organizational factors that consistently emerged and were supported by a substantial body of evidence with relation to outbreak include staffing levels [26,28,34,48,56,59,70,72,76,79,80,82,84,91,92,94,97,99,106,113], star/subset domains ratings [59,63,69,71,74,76,79,82,83,88,89,94,99,106,111,113], LTCFs with a higher proportion of racial and ethnic minorities [67,70,73,77,81,83,84,89,94,95,99,101,105,106,109], type of ownership (for-profit facilities) [6,24,26,30,37,57,63,72,79,82,84,89,97,113], LTCFs with higher Medicaid-insured residents [71,72,73,91,94,95,97], presence of infected staff [36,42,47,54,56,89,113], quality performance [31,34,43,71,106], and chain membership status [21,30,70,97].

Table S5 contains details about the organizational factors associated with COVID-19 outbreaks in LTCFs.

#### 3.2.2. Organizational Factors Related to COVID-Related Deaths

COVID-related deaths were associated with six organizational areas that we further categorized into five groups: (1) LTCFs quality indicators (star rating, quality performance), (2) staffing (staffing levels, infected staff), (3) ownership and chain affiliation (ownership types, chain membership status), (4) Medicaid and Medicare coverage, and (5) LTCF’s racial and ethnic composition.

The organizational factors that consistently emerged and were supported by a substantial body of evidence with relation to mortality include LTCF’s racial and ethnic composition [2,63,67,70,75,81,83,85,89,95,96,103,109,110], for-profit and/or private ownership [2,24,30,56,63,83,87,98,102,104,113], nursing staffing levels [2,48,56,75,80,81,92,94,98,110], star rating and subdomains (inverse association) [63,74,81,87,88,98,111,113], infected staff [27,56,66,89,113], chain membership [33,70,75,102,104], quality performance [29,40,62,75].

Table S6 depicts detailed information about the impact of organizational factors on COVID-19-related death.

### 3.3. Environmental Factors

Environmental factors were conceptualized as the social and physical environments in which residents reside.

#### 3.3.1. Environmental Factors Related to COVID Outbreaks

Two groups of environmental factors covering 13 areas were found to affect COVID-19 outbreaks: (1) community factors (outbreaks in counties/communities, outbreak in the community where staff live, location of LTCF, community sociodemographic status (e.g., racial/ethnic composition of the community and socioeconomic status), and (2) physical characteristics (number of beds, crowding index, occupancy rate, new admissions, structural design of the rooms, using the Green House model, accessing ventilator-dependent units, mechanical recirculation of air, and type of care provided in the ward.

The environmental factors frequently emerged and supported by a substantial body of evidence linked to outbreaks include a higher number of beds, occupancy rate [21,25,26,30,31,34,47,54,56,60,61,63,65,67,72,73,82,83,84,91,94,97,106], presence of outbreaks in surrounding counties/communities [6,26,29,30,31,37,38,60,69,79,80,84,91,93,99,104], high-density communities [21,29,56,67,73,106] socioeconomic status of the community, [24,64,77,84,97,106], the structural design of the rooms [21,25,26,104,113], the racial/ethnic composition of the community [63,70,84,113], the location of the LTCF [21,24,43,56], older design and facility age [30,31,56,83].

Detailed information on these environmental factors is provided in Table S7.

#### 3.3.2. Environmental Factors Related to COVID-Related Deaths

Ten environmental factors associated with COVID-19-related death were identified and further categorized into two groups: (1) community factors (outbreaks in counties/communities, high-density communities, public transportation use by workers, disability support program use, location of LTCF, community sociodemographic status (proportions of ethnic minorities and socially deprived communities), and (2) LTCFs physical characteristics (bed numbers, levels of crowding, structural design of the rooms, using the Green House model, accessing ventilator-dependent unit).

The environmental factors frequently associated with mortality include occupancy, levels of crowding [2,25,56,64,83,94,98,104], the larger size of the facility or the number of beds in the LTCF [2,32,33,62,63,65], the location of the LTCF [2,21,24,62,87], higher community or county incidences [60,77,80,104,113], and structural design of the rooms or number of beds in a room [21,25,66,113].

Table S8 contains detailed information about the impact of organizational factors on COVID-19-related death.

## 4. Discussion

The COVID-19 pandemic led to excess deaths of older adults in LTCFs, particularly early on, when there were few effective treatments and no vaccination programs [117]. This, coupled with staffing shortages, and the relative shortages of adequate personal protective equipment [118] exacerbated the already parlous state within LTCFs.

**Figure 2:**
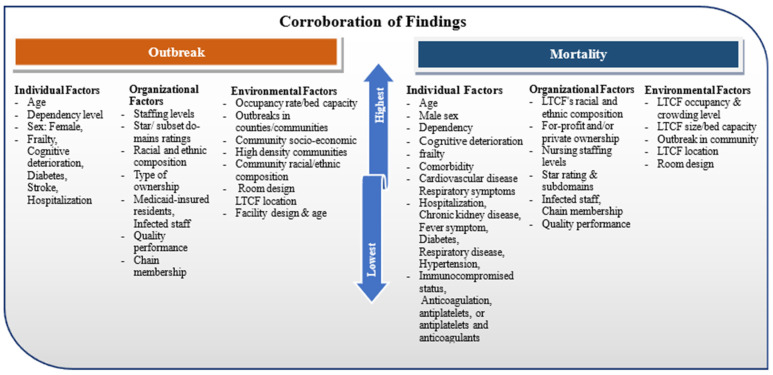
Individual, organizational, and environmental factors affecting COVID-19 outcomes.

There is an abundance of information on the contributors to outbreaks and deaths. In looking across the included citations, the most influential factors begin to emerge. Figure 2 depicts factors with the greatest support.

Among the individual factors identified in contributing to outbreaks were a larger representation of older age, increased resident dependency, sex (female), and higher Frailty Index, as well as presence of comorbidities and cognitive decline/dementia.

Arranging individual factors based on the strongest research support reveals the following sequence of their influence on mortality: comorbidity, in particular older age, male sex, increased dependency, cognitive deterioration/dementia, and frailty levels, and co-morbidities.

The individual factors are unsurprising. The population residing in LTCFs represents the most vulnerable older adults, with complex comorbidities and a high prevalence of Alzheimer’s disease and related dementias. The greater likelihood of outbreaks in those exhibiting responsive behaviours is predictable, given that adherence to the strict infection control required is more difficult for these individuals and their dependency upon staff much greater. Cognitive impairment was the most extensively researched comorbidity and a significant predictor of COVID-related outcomes in LTCFs. This result ties well with a systematic review and meta-analysis [119,120]. Underlying mechanisms identified as being implicated in the increased risk were neuroinflammation, nonadherence to COVID-19 prevention measures, and the impact of coexisting comorbidities [119,120].

When looking at organizational factors, parallels emerged for outbreaks and mortality. For outbreaks, staffing levels (an inverse association), star and subset domain ratings for the facility, higher percentages of racial and ethnic minorities in LTCFs, ownership types (including private, for-profit, and chain membership), and presence of infected staff were corroborated across studies. The organizational factors with the highest level of research support for mortality were similar but with a slightly different rank order: LTCF’s racial and ethnic composition, private/for-profit ownership and chain membership, staffing levels, star and subset domain ratings, and the presence of infected staff. A lower quality of LTCF care has previously been linked to for-profit status ownership [121], and perhaps this finding illustrates the same paradigm.

Unsurprisingly, staffing levels, which also may be lower in private and for-profit homes, and which were further stressed during the pandemic, were identified as a significant risk in outbreak and death analyses. A higher risk of adverse COVID-19 outcomes was identified within LTCFs having larger proportions of racial/ethnic minority residents; it was noted that the disparities in nursing home outcomes attributed to race were not solely a result of race. Instead, these disparities were rooted in underlying inequalities inherent in healthcare and nonhealthcare sectors, ultimately leading to poorer health outcomes for racial/ethnic minority residents [89,95].

Not all studies attempted to control for socioeconomic factors underlying ethnic differences in susceptibility, either that of the residents, their care staff, or the socioeconomic status of the facility or of the community in which it resided, exposing the inter-relatedness of the three strata analyzed.

The variability regarding the influence of staffing levels can be attributed to three main factors. Firstly, discrepancies or challenges in methods and study quality may play a role, as different studies utilized diverse analysis techniques and might have overlooked adjusted analyses. This aligns with the findings of Harrington and colleagues [82], who found that low total nurse staffing hours were associated with outbreaks when the model was not adjusted for factors, including health deficiencies, bed size, ownership, and total nurse staffing hours. Adjusting for multi-level factors might have yielded different results. Studies also used various databases to gain information about the characteristics of the LTCFs in varying time periods before the pandemic; these might not reflect the characteristics of the LTCF staff attrition that occurred due to COVID-19 [122]. Furthermore, data related to the COVID-19 outcomes were often collected in different time frames, with little attention to longitudinal as the pandemic progressed. Methodological challenges may also arise in connection with interpretive analysis, leading to divergent results. For example, when interpreting staff–resident ratios, discrepancies may occur based on whether the numerator considers solely full-time staff or encompasses casual workers, as noted in a distinct systematic review [41]. Secondly, concerning differences in staffing policies, exemplified by variations in pre-COVID-19 staffing regulations governing casual and part-time employment or work across multiple LTCFs, could potentially contribute to the transmission of diseases [123]. Thirdly, contextual factors may contribute to the variation in the effects of staffing levels on outcomes; results could be influenced by whether the staff members come from communities experiencing active outbreaks, thereby affecting the potential for disease transmission within LTCFs [73]. The availability of personal protective equipment (PPE) and consistent infection control and prevention training throughout the pandemic could also shape outcomes [91].

Given the conflicting data regarding the effects of staffing. a more comprehensive investigation is warranted. This could involve retrospective longitudinal studies that span the entirety of the pandemic, utilizing repeated measures designs. Additionally, utilizing time-sensitive data related to COVID-19 outcomes and the specific attributes of the facilities under study would provide a more comprehensive understanding of the impact. To capture the whole picture of a phenomenon, performing an adjusted analysis by including the individual, organizational, and environmental covariates would be required.

The environmental factors that garnered the most substantial support regarding outbreaks and mortality alike were the number of beds/crowding index/occupancy rate, outbreaks in counties/communities, community sociodemographic status (racial/ethnic composition of the community/socioeconomic status), high-density communities and structural design of the rooms. Similarly, the strongest environmental factors contributing to COVID-19-related mortality included the number of beds/crowding index/occupancy rate, outbreaks in counties/communities, location of the LTCF, and structural design of the rooms.

A scoping and systematic review demonstrated that certain studies show a relationship between the for-profit status of care facilities and related attributes, like sufficient staffing, access to personal protective equipment (PPE), and testing provisions. These factors are then tied to increased adverse COVID-19 outcomes. The reviews also indicated that the influence of ownership is intricate and holds significance [124,125].

The higher occupancy metrics (including number of beds, crowding index, occupancy rate) and occurrences of outbreaks in counties and communities where LTCFs were located were predominant environmental factors, which is aligned with a previous systematic review wherein the congregate physical environment in LTCFs was described as a factor that exacerbates the outbreaks and mortality risk [10]. Larger LTCF size, location, and interaction with the community with high COVID-19 rates were described as the strongest and most consistent predictors of COVID-19 outcomes [10].

These findings illustrate the inter-relatedness of the classification of strata, homes in poorer neighbourhoods, drawing their staff from less privileged communities (all factors associated with outbreaks) may also have residents who similarly themselves have a high proportion of “at risk” factors. A lack of single rooms, making isolation in the face of infection more difficult, is perhaps a predictable finding, as is the existence of high-density living and older home design (perhaps more “institutional”). Findings such as those in the “Green House” model (smaller, more home-like communities) are perhaps less expected but again may illustrate resident-based risk, rather than an institutional factor.

Discrepancies within articles regarding both COVID-19 outbreaks and mortality may be partially attributed to outcome reporting bias. Notably, clarity is absent regarding whether the prevalence of COVID-19 encompasses asymptomatic residents as well or only those confirmed with tests. Similarly, for mortality rates it remains uncertain whether deaths among residents with positive COVID-19 status died because of, or with, COVID-19. This was evident in a study where 22.7% of COVID-19 cases resulted in death, of which only 24.8% were classified as COVID-19-related deaths [66].

Our study does have limitations. Observational studies are prone to biases, such as reverse causation and residual confounding [126]. The appraisal tool used for this study may not explicitly focus on issues like reverse causality or other forms of endogeneity in observational studies.

We did not include studies focusing mainly on residents transferred to the hospital to treat COVID-19 infection or those that implemented interventions that could be considered organizational. Included study designs were observational, describing strength of association rather than allowing inference of causality. Analyses also failed to consider the complexity of the interrelationship between factors; of the studies included here, only 40% took into account individual, organizational, and environmental (both internal and external to the facility) factors collectively, all including varying covariates/cofounders. Conversely, around 30% of the articles focused solely on one of these three strata, potentially introducing confounding effects due to unmeasured variables.

Many studies did not provide a fully adjusted analysis, which could have reduced the bias in the parameter estimates, so the results might have been overestimated. There was no consistent pattern of adjustment in analytical models across the papers, which may have affected the results. The sample size in seven studies was small and included only one to six LTCFs. More than half of the studies took place in the USA, with data from five studies obtained from one state. This may introduce a systematic bias, being based upon a common administrative structure. The remaining locations form an unrepresentative sample of international LTCF.

Furthermore, non-English papers were not included in this review; thus, a small percentage of the overall number of relevant articles have been excluded. We were also unable to take an intersectional lens in analyzing social determinants of the health of residents, their care providers, and the communities in which the LTCFs were situated. Some of the factors identified in this review are supported by very little evidence, requiring further studies. Furthermore, few LTCF care delivery models were covered, and the Green House model was the focus of only one study.

The results of this study offer comprehensive insights into the complexities of the phenomena and may support the development of modeling which incorporates the multi-level nature of factors influencing outcomes. The findings may also inform decision-makers about those that need to be taken into account to mitigate the impact of this and future pandemics in LTCFs. We have highlighted areas that have not been rigorously researched and those factors that can be considered covariates to control for in future analyses. We also encourage using concurrent data as the validity of the study’s results may be compromised if using non-contemporary data (e.g., two-year-old data regarding characteristics of the organizations).

## 5. Conclusions

This review has identified potentially modifiable individual, organizational, and environmental risk factors for COVID-19-related outbreaks and deaths in LTCFs for older adults. Action to address these factors is a matter of urgency. To address the risk factors identified in this systematic review, several important actions are recommended. Initially, focus should be devoted to enhancing staffing levels by employing recruitment of full-time staff and training programs to ensure that residents receive sufficient support and mitigating the risk of cross-contamination and transmission within the facility by proactively developing proper strategies. Furthermore, improving quality rankings and performance standards may enhance the overall quality of care. Addressing disparities and racial and ethnic barriers to effective healthcare services in LTCFs and communities requires strategic interventions to mitigate underlying healthcare inequalities. To address specific challenges for residents with dementia in LTCFs, specific plans need to be developed. Initiatives for community engagement and support can enhance resources and tackle social determinants of health. Lastly, prioritizing further research to gain a deeper understanding of these complex interactions will inform evidence-based interventions and policies for managing future pandemics.

In light of the factors identified, including age, sex, dependency level, dementia prevalence, quality performance metrics, staffing levels, racial compositions within LTCFs, ownership structure, bed count, occupancy rate, and community/county characteristics, we recommend a meta-analysis that includes more comparisons to estimate the effectiveness of these factors on COVID-19 outcomes in LTCFs.

## Figures and Tables

**Table 1:** Description of studies included in the systematic review.

First Author	Year	Time Frame Data Collection	Country/ Region	Outcome Type	Objectives	LTCF #	Study Design	Factors’ Level	AA/EA
Heras [20]	2021	15 March 2020 to 5 June 2020	Andorra	M	Identification of the COVID-19 mortality risk factors in older people from a long-term care center	-	Cross-sectional	Indiv	+
Ibrahim [21]	2021	7 July to 13 November 2020	Australia/Victoria	O and M	Investigation of the LTCFs characteristics, including organizational and facility structures, location, and access to acute health care, associated with the COVID-19 outbreak	766	Cross-sectional	Org, Envi	-
Peckeu-Abboud [22]	2022	8 April 2022, to 15 May 2020	Belgium	O	Identification of the factors influencing SARS-CoV-2 infection rate in Belgian LTCFs residents during the first wave of the COVID-19 pandemic	695	Cross-sectional	Indiv, Org	+
Frigotto [23]	2023	5 November to 31 December 2020	Brazil	O	Investigation of whether the functional capacity prior to COVID-19 infection was different between survivor and non-survivor older adults	2	Cross-sectional	Indiv	-
Akhtar -Danesh [24]	2022	January 2019 to December 2020	Canada/Ontario	O and M	Determination of the scale of pandemic-related deaths of long-term care residents in the province of Ontario, Canada, and to estimate excess mortality due to a positive COVID-19 test adjusted for demographics and regional variations	626	Cohort	Indiv, Org, Envi	+
Brown [25]	2021	29 March 2020 to 20 May 2020	Canada/Ontario	O and M	Determination of whether crowding was associated with COVID-19 cases and mortality in the first months of the COVID-19 epidemic	618	Cross-sectional	Indiv, Org, Envi	+
Cox [26]	2023	1 March 2020, to 31 January 2021	Canada/British Columbia	O and M	Assessment of whether facility ownership was associated with COVID-19 outbreaks among LTCFs	293	Cross-sectional	Org, Envi	+
Fisman [27]	2020	March 2020 to April 2020	Canada/Ontario	O and M	Identification of the trends and risk factors associated with COVID-19 death in LTCFs in Ontario, Canada	627	Cohort	Org, Envi	+
Kain [28]	2021	1 March to 21 May 2020	Canada/Ontario	O	Understanding of the way in which the virus spreads within these homes is critical to preventing further outbreaks.	1	Cross-sectional	Indiv, Org, Envi	-
Lee [29]	2021	1 January to 31 August 2020	Canada/Ontario	M	Understanding of the contributions to mortality among SARS-CoV-2-infected residents in LTC homes	-	Cross-sectional	Indiv	+
Stall [30]	2020	29 March to 20 May 2020	Canada/Ontario	O and M	Examination of the association between for-profit status and the risk of COVID-19 outbreaks and death during the peak of the epidemic in Ontario’s LTC homes	623	Cross-sectional	Org, Envi	+
Vijh [31]	2021	1 March 2020, to 10 January 2021	Canada/British Columbia	O	Identification of the risk factors associated with outbreak severity to inform current outbreak management and future pandemic preparedness planning efforts	48	Cross-sectional	Indiv, Org, Envi	+
Zhang [32]	2022	23 February to 11 July 2020	Canada/Montreal	M	Identification of the individual, therapeutic, and institutional factors associated with death in LTCFs	17	Cross-sectional	Indiv, Org	+
Morciano [33]	2021	1 January 2017, to 7 August 2020	England	M	Investigation of the excess mortality for care home residents during the COVID-19 pandemic in England, exploring associations with care home characteristics	4428	Cohort	Indiv, Org, Envi	+
Shallcross [34]	2021	26 May to 19 June 2020	England	O	Identification of the factors associated with SARS-CoV-2 infection and outbreaks among staff and residents in LTCFs	5126	Cross-sectional	Indiv, Org, Envi	+
Corvol [35]	2022	1 March to 31 May 2020	France/Brittany	O	Identification of the structural and managerial factors associated with COVID-19 outbreaks in LTCFs	231	Cross-sectional	Indiv, Org, Envi	+
Piet [36]	2021	1 March to 31 May 2020	France/French Alps	O and M	Identification of the relationship between the occurrence of an outbreak of COVID-19 among residents and staff members	225	Cross-Sectional	Indiv, Org, Envi	+
Rabilloud [37]	2023	1 March to 31 July 2020, and 1 August to 31 December 2020	France/Auvergne-Rhône-Alpes	O and M	Quantification of the effects of characteristics of LTCFs and their surroundings on the spread of COVID-19 outbreaks and assessment of the changes in resident protection between the first 2 waves	937	Cross-sectional	Org, Envi	+
Rabilloud [38]	2022	1 March to 31 July 2020	France/Auvergne-Rhône-Alpes	O	Identification of the role SARS-CoV-2 virus spread in nearby population plays in introducing the disease in LTCFs	943	Cross-sectional	Indiv	-
Sacco [39]	2020	Prior to 17 March 2020	France/Maine-et-Loire	O	Comprehensive description of the symptoms and chronological aspects of the diffusion of the SARS-CoV-2 virus in an LTCF, among both residents and caregivers	1	Cross-sectional	Indiv	+
Tarteret [40]	2021	18 March to 10 April 2020	France/Ile-de-France	M	Identification of the demographic, clinical, and medical care factors associated with mortality in LTCF residents	3	Cross-sectional	Indiv, Org	+
Preuß [41]	2023	28 April to 12 May 2020 and 12 January to 7 February 2021	Germany	O and M	Identification of the impact of Facilities’ Structures on the Morbidity and Mortality of Residents	824 and 385 during 1st and 2nd waves, respectively	Cross-Sectional	Org, Envi	+
Kennelly [42]	2021	29 February to 22 May 2020	Ireland	O and M	Examination of characteristics of LTCFs across three Irish Community Health Organisations, proportions with COVID-19 outbreaks, staff and resident infection rates, symptom profile and resident case fatality	45	Cross-Sectional	Indiv, Org, Envi	-
Cazzoletti [43]	2021	March to May 2020	Italy/Trento	O	Examination of the association between certain measurable factors (structural, organizational, and practice-related) and the cumulative incidence of COVID-19 among LTCF residents in the Autonomous Province of Trento, Italy, during the peak of the COVID-19 outbreak	57	Cross-sectional	Indiv, Org, Envi	+
Veronese [44]	2021	1 March to 31 December 2020.	Italy/Venice	M	Examination of whether COVID-19 was associated with a higher mortality rate in LTCF residents, considering frailty status assessed with the Multidimensional Prognostic Index	31	Cross-sectional	Indiv	+
Arienti [45]	2021	1 March to 7 May 2020	Italy/Lombardy	O and M	Description of the epidemiological characteristics of LTCF residents infected by severe acute respiratory syndrome coronavirus 2 (SARS-CoV-2) infection and to compute the related case-fatality rate	6	Cross-sectional	Indiv	-
Cangiano [46]	2020	March to April 2020	Italy		Quantification of the impact of SARS-CoV-2 on mortality in LTCFs and to point out the factors related to its severity as well as the limitations of diagnostic tests used to manage the spread of this infective disease	1	Prospective	Indiv	-
Orlando [47]	2022	March to December 2020	Italy/Lazio	O	Understanding of which organizational–structural characteristics of LTCFs associated with the risk of a COVID-19 outbreak	100	Case–control	Org, Envi	+
Lee [48]	2022	20 January to 20 October 2020	Korea	O and M	Examination of the organizational factors and characteristics of NH related to the COVD-19 outbreak and mortality during the COVID-19 pandemic’s peak	3389	Cross-sectional	Org	+
Booij [49]	2022	March to November 2020	Netherlands	O and M	Investigation of short- and long-term mortality and risk factors in LTCFs patients with COVID-19	2	Cross-sectional	Indiv	+
Houben [50]	2023	September 2020 to June 2021	Netherlands/South Limburg	O	Identification of the facility- and ward-level factors associated with SARS-CoV-2 outbreaks among LTCF residents	60	Cross-sectional	Org, Envi	+
Rutten [51]	2020	18 March to 13 May 2020.	Netherlands	O and M	Description of the symptomatology, mortality, and risk factors for mortality in a large group of Dutch LTCF residents with clinically suspected COVID-19 who were tested with a reverse transcription–polymerase chain reaction (RT–PCR) test	NI	Prospective	Indiv	+
Visser [52]	2023	March 2020 to December 2021	Netherland	M	Identification of the impact of polypharmacy on 30-day COVID-related mortality after adjustment for age, sex, CCI, BMI, and vaccination status	15	Cross-sectional	Indiv	+
Arendse [53]	2022	5 March 2020 to 31 July 2022	South Africa	M	Description of the temporal trends as well as the characteristics and risk factors for mortality	45	Cross-sectional	Indiv	+
Agoües [54]	2021	15 March to 15 May 2020	Spain/Sant Cugat del Vallès	O and M	Identification of the factors related to morbidity and mortality of COVID-19	12	Cross-sectional	Indiv, Envi	+
Aguilar-Palacio [55]	2022	9 March 2020 to 14 March 2021	Spain/Aragón	O and M	Description of the profile of institutionalized patients with a confirmed COVID-19 infection and the socioeconomic and morbidity factors associated with hospitalization and death	1	Cross-sectional	Indiv	+
Arnedo-Pena [56]	2022	March 2020 to January 2021	Spain/Castellon	O and M	Investigation of the incidence and mortality of COVID-19 in LTCFs, and their associated risk factors, before starting the vaccination against the disease	27	Cross-sectional	Indiv, Org, Envi	+
Escribà-Salvan [57]	2022	December 2019 to March 2021	Spain/Central Catalonia	O	Identification of the risk factors associated with developing COVID-19 infection with symptoms in institutionalized older people	5	Longitudinal	Indiv, Org	+
Romero [58]	2020	March 2020 to 5 April 2020	Spain/Albacete	O and M	Investigation of the mortality, costs, residents, and personnel characteristics in six long-term care facilities (LTCFs) during the outbreak of COVID-19 in Spain	6	Cross-sectional	Indiv	-
Meis-Pinheiro [59]	2021	1 March to 31 May 2020	Spain/Catalonia	O	Identification of the clinical characteristics of COVID-19 in older adults in LTCFs	80	Cross-sectional	Indiv	+
Soldevila [60]	2021	1 March to 30 June 2020	Spain/Barcelona in Catalonia	O and M	Identification of the interplay between infection risk factors of SARS-CoV-2 and prognosis	168	Cross-Sectional	Indiv, Envi	+
San Román [61]	2023	July to December 2020	Spain/Madrid	O	Identification of the factors that facilitate COVID-19 outbreaks in LTCFs	369	Cross-sectional	Indiv, Org	+
Suñer [62]	2021	March to 1 June 2020,	Spain/Catalonia	M	Investigation of the determinants of mortality in LTC facilities during the COVID-19 outbreak	167	Cross-sectional	Indiv, Org, Envi	+
Telleria [63]	2022	March to December 2020	Spain/Guipúzcoa	O and M	Identification of the association of demographic, clinical, and pharmacological risk factors with the COVID-19 infection, and related death	4	Case–control	Indiv	+
Torres [64]	2022	March to June 2020	Spain/Barcelona	O and M	Identification of the inequalities in the cumulative incidences (CIs) and in the mortality rates (MRs) due to COVID-19	232	Longitudinal	Org, Envi	+
Zunzunegui [65]	2022	March to April 2020	Spain/Catalonia	O and M	Understanding how COVID-19 infection and mortality varied according to facility size	965	Cross-Sectional	Org, Envi	+
Scanferla [66]	2023	February 2020 to 31 May 2021	Swiss	O and M	Identification of the influencing factors for a higher COVID-19 burden	59	Cross-sectional	Indiv, Org, Envi	+
Abrams [67]	2020	By 11 May 2020	USA	O	Examination of the characteristics of LTCFs with documented COVID-19 cases in 30 states reporting individual facilities affected	9395	Cross-Sectional	Indiv, Org, Envi	+
Adler [68]	2022	1 March 2020, and 31 May 2020	USA	M	Evaluation of the impact of the underlying use of antithrombotic on the 30-day mortality of individuals with COVID-19 and to assess the relationship between age and sex with 30-day all-cause mortality in patients with COVID-19	NI	Cross-sectional	Indiv	+
Bagchi [6]	2021	25 May to 22 November 2020	USA	O	Identification of the rates of COVID-19 among residents and staff members in LTCFs	15,342	Cross-sectional	Org, Envi	-
Bui [69]	2020	17 March to 11 June 2020	USA/West Virginia	O	Identification of the relationship of the quality of LTCFs (using star ratings) and COVID-19 outbreak	123	Cross-Sectional	Org, Envi	+
Cai [70]	2021	7 June 2020 to 23 August 2020	USA	O and M	Examination of the racial and ethnic composition of LTCFs and communities with relation to the COVID-19 cases and death in LTCFs	13,123	Cross-sectional	Indiv, Org, Envi	+
Chatterjee [71]	2020	22 April to 29 April 2020	USA/23 states and the District of Columbia	O	Description of the characteristics and quality of LTCFs with COVID-19 cases in states where public health departments have begun to publicly report their statuses	8943	Cross-sectional	Org, Envi	-
Chen [72]	2021	By 11 October 2020	USA	O	Identification of association between LTCF characteristics and resident COVID-19 morbidity in communities with high infection rates	2017	Cross-sectional	Indiv, Org, Envi	+
Chen [73]	2020	13 April to 23 August 2020	USA	O	Investigation of LTCF staff networks and COVID-19	13,165	Cross-sectional	Indiv, Org, Envi	+
Cooper [63]	2021	June 2020 to January 2021	USA	O and M	Determination of whether LTCFs COVID-19 patient outcomes varied from one nursing facility to another based on location and resident characteristics, and the availability of COVID-19-specific medical equipment in the LTCFs impacts morbidity and mortality outcomes.	14,405	Cross-sectional	Indiv, Org, Envi	+
Cronin [74]	2022	May 2020 to September 2021	USA	O and M	Investigation of whether high-quality LTCFs measured by the CMS five-star ratings did a better job of preventing deaths from COVID-19	14,905	Cohort	Org, Envi	+
Dean [75]	2020	1 March to 31 May 2020	USA/New York	O and M	Identification of the association between the presence of healthcare worker unions and additional facility-level factors and COVID-19 mortality rates	355	Cross-sectional	Indiv, Org, Envi	+
Figueroa [76]	2020	1 January to 30 June 2020	USA/8 states	O	Evaluation of whether LTCFs, rated highly by the Centers for Medicare & Medicaid Services (CMS) across 3 unique domains—health inspections, quality measures, and nurse staffing—had lower COVID-19 cases than facilities with lower ratings	4254	Cross-sectional	Org, Envi	+
Engeda [77]	2023	25 May to 16 August 2020	USA/California	O	Identification of the association between LTCFs COVID-19 infections and level of racial and ethnic compositions and facility- and neighborhood-level (census tract- and county-level) indicators of socioeconomic status	971	Cross-sectional	Indiv, Envi	+
Gilman [78]	2021	25 May 2020, to 18 April 2021	USA	M	Identification of the trends in COVID-19 death rates by the racial composition of LTCFs through mid-April 2021	13,820	Cross-sectional	Indiv	-
Gopal [79]	2021	1 May 2020	USA/California	O	Understanding of why some LTCFs are more susceptible to larger COVID-19 outbreaks.	713	Cross-sectional	Org, Envi	+
Gorges [80]	2020	25 June 2020	USA	O and M	Understanding of whether baseline nurse staffing is associated with the presence of COVID-19 in LTCFs and whether staffing impacts outbreak severity	13,167	Cross-sectional	Indiv, Org, Envi	+
Gorges [81]	2021	1 January 2020, to 13 September 2020	USA	M	Description of differences in the number of COVID-19 deaths by LTCFs racial composition and examine the factors associated with these differences	13,312	Cross-sectional	Indiv, Org, Envi	+
Harrington [82]	2020	March to 4 May 2020	USA/California	O	Examination of the relationship of nurse staffing in California LTCFs and compare homes with and without COVID-19 residents	1091	Cross-sectional	Org, Envi	+
He [83]	2020	2 June 2020	USA/California	O and M	Determination of whether COVID-19 cases and deaths are related to the LTCFs reported quality	1223	Cross-sectional	Indiv, Org, Envi	+
Hege [84]	2021	1 June 2020, to 31 January 2021	USA	O	Investigation of the relationship between US LTCF-associated COVID-19 infection rates and county-level and LTCFs attributes	9990	Cohort	Indiv, Org, Envi	+
Hill [85]	2022	March 2020 to March 2021	USA/Alameda	M	Identification of the differential impacts across settings as well as racial and economic disparities among long-term care residents with relation to COVID-19-related death	592	Cross-sectional	Indiv	-
Hua [86]	2022	12 March to 31 December 2020 and 1 January 2019, to 11 March 2020	USA	M	Comparison of the weekly rate of excess all-cause mortality during the first several months of the COVID-19 pandemic among residents with ADRD in assisted living to those without ADRD and among residents with ADRD in memory care assisted living or in general assisted living	NI	Cross-sectional	Indiv	+
Iyanda [87]	2022	1 January 2020 to 18 December 2021	USA	M	Examination of the determinants of COVID-19 deaths in LTCFs in the first 2-year pandemic period	13,350	Cross-sectional	Org, Envi	+
Khairat [88]	2021	25 May to 20 December 2020	USA	O and M	Identification of the relationship between LTCFs’ quality and the spread and severity of COVID-19 in LTCFs	15,390	Cross-sectional	Org, Envi	+
Kim [89]	2022	1 January to 30 September 2021	USA/Illinois	O and M	Examination of the pathways through which community and facility factors may have affected COVID-19 cases and deaths	177	Cross-sectional	Indiv, Org, Envi	+
Kumar [90]	2021	1 June to 27 December 2020	USA	O and M	Examination of the impact of a high proportion of minority residents in LTCFs on COVID-19-related mortality rates over a 30-week period.	11,718	Longitudinal	Indiv, Org, Envi	+
Lane [91]	2022	May to September 2020, September to December 2020, and December to February 2021	USA-Southern	O	Identification of the LTCFs and county-level predictors of COVID-19 outbreaks in LTCFs in the southeastern region of the United States across three time periods.	2951	Longitudinal	Indiv, Org, Envi	+
Levy-Storms [92]	2022	1 January 2020 to 2 July 2021	USA	O and M	Determination of the role of Certified Nursing Aides (CNAs) in the care of residents living in LTCFs	-	Cross-sectional	Org	-
Longo [93]	2022	June 2020 to January 2021	USA/Illinois, Florida, and Massachusetts	O	To compare COVID-19 cases among accredited and nonaccredited LTCFs		Cross-sectional	Envi	+
Li [94]	2020	By 16 April 2020	USA	O and M	Determination of the associations of LTCFs registered nurse (RN) staffing, overall quality of care, and concentration of Medicaid or racial and ethnic minority residents with 2019 coronavirus disease (COVID-19) confirmed cases and deaths	215	Cross-sectional	Indiv, Org, Envi	+
Li [95]	2020	25 May to 31 May 2020	USA	O and M	Determination of the racial/ethnic disparities in weekly counts of new COVID-19 cases and deaths among LTCFs residents or staff	12,576	Cross-sectional	Indiv, Org, Envi	+
Li [96]	2022	13 April to 19 June 2020	USA	O and M	Evaluation of the trends in racial and ethnic disparities in weekly cumulative rates of coronavirus disease 2019 (COVID-19) cases and deaths	211	Longitudinal	Indiv	+
Lord [97]	2021	1 January 2020 to 11 July 2021	USA	O	Examination of the relationship between community resource scarcity, as conceptualized by the Social Deprivation Index (SD), and COVID-19 incidence rates in LTCFs.	13,772	Cross-sectional	Indiv, Org, Envi	+
Lu [98]	2021	1 April 2020 to 22 December 2020	USA	M	Evaluation of risk factors for COVID-19 deaths and hospitalizations among LTCFs Medicare beneficiaries in the prevaccine pandemic period.	-	Cross-sectional	Indiv, Org, Envi	+
Mattingly [99]	2021	1 January to 1 July 2020	USA/Maryland	O	Identification of the characteristics associated with large outbreaks	216	Cross-sectional	Indiv, Org, Envi	+
McGarry [100]	2021	By 30 September 2020	USA	O and M	Examination of how the number of unique staff members might influence the likelihood of COVID-19 cases and deaths in LTCFs	15,071	Cross-sectional	Indiv, Org, Envi	+
Mehta [101]	2021	1 April to 30 September 2020	USA	O and M	Identification of the risk factors for SARS-CoV-2 incidence, hospitalization, and mortality among LTCF residents in the US	482, 323	Cross-sectional	Indiv	+
Olson [102]	2022	24 April 2022	USA	O and M	Understanding of the association between LTCFs unionization and resident COVID-19 mortality percentage	14,380	Cross-sectional	Indiv, Org, Envi	+
Panagiotou [103]	2021	16 March to 15 September 2020	USA	M	Identification of the risk factors for 30-day all-cause mortality among US LTCF residents with COVID-19.	5256	Cross-sectional	Indiv	+
Shen [104]	2022	5 July–10 July 2020	USA/18 states	M	Examination of the relationship between LTCFs COVID-19 outbreaks and differences in the characteristics of the residential neighbourhoods	6132	Cross-sectional	Indiv, Org, Envi	+
Shi [105]	2020	16 March to 8 May 2020	USA/Boston, MA	O and M	Description of the clinical characteristics and risk factors associated with coronavirus disease 2019 (COVID-19) in long-stay LTCF residents	1	Cross-sectional	Indiv	+
Sugg [106]	2021	By 30 June 2020	USA	O and M	Determination of the association between LTCF-level metrics and county-level, place-based variables with COVID-19 confirmed cases in LTCFs across the United States	13,709	Cross-sectional	Org, Envi	+
Tang [107]	2020	1 March to 12 June 2020	USA/Maryland	M	Identification of the association of symptom status and medical comorbidities on mortality and hospitalization risk associated with COVID-19	15	Cross-sectional	Indiv	+
Thorsness [108]	2022	1 March to 31 December 2020	USA	M	Identification of the association of kidney function with 30-day mortality following COVID-19 infection	176	Cross-sectional	Indiv	+
Travers [109]	2021	20 January to 19 July 2020	USA	O and M	Examination of the associations between the proportion of Black residents in LTCFs and COVID-19 infections and deaths, accounting for structural bias (operationalized as county-level factors) and stratifying by urbanicity/rurality	11,587	Cross-sectional	Indiv, Org, Envi	+
Unruh [2]	2020	20 January–30 April 2020	USA/Connecticut, New Jersey, and New York	M	Evaluation of the characteristics of LTCFs with COVID-19 deaths compared to other LTCFs using data from Connecticut, New Jersey, and New York	1162	Cross-sectional	Indiv, Org, Envi	+
Weech-Maldonado [110]	2021	1 January to 25 October 2020	USA	M	Examination of the relationship between LTCFs racial/ethnic mix and COVID-19 resident mortality	12,914	Cross-sectional	Indiv, Org, Envi	+
Williams [111]	2021	10 January 2021	USA	O and M	Understanding of the relationship of LTCFs quality ratings and measures of COVID-19 outbreak severity and persistence	14,693	Cross-sectional	Indiv, Org, Envi	+
Young [112]	2023	31 May 2020 to 27 March 2022	USA/New York	O and M	Evaluation of the COVID-19 case and mortality rates in green houses compared to traditional LTCFs in New York state	608	Cross-sectional	Org	-
Zhu [113]	2022	7 June to 20 December 2020	USA	O and M	Examination of the associations of LTCFs design with COVID-19 cases, deaths, and transmissibility and provide relevant design recommendations	7785	Cross-sectional	Indiv, Org, Envi	+
Zimmerman [114]	2021	20 January to 31 July 2020	USA	O and M	Comparison of the rates of COVID-19 infections, COVID-19 admissions/readmissions, and COVID-19 mortality, among Green House/small LTCFs with rates in other LTCFs	43	Cross-sectional	Org	-
Emmerson [115]	2022	1 September to 31 December 2020	Wales	O	Identification of the impact of dementia, frailty, and care home characteristics on SARS-CoV-2 incidence in a national cohort of Welsh care home residents during a period of high community prevalence	673	Cross-sectional	Indiv, Org, Envi	-

Abbreviation: NI: Not identified, M: Mortality, O: Outbreak; ADRD: Alzheimer’s disease and related dementias, Envi: Environmental, Indiv: Individual; Org: Organizational; AA/EA: Adjusted analysis/Effect analysis, ‘+’ symbol indicates presence of Adjusted Analysis/Effect Analysis and ‘-’ symbol indicates absence.

## Data Availability

Data are contained within the article and Supplementary Materials.

## Supplementary Materials

The following supporting information can be downloaded at: https://www.mdpi.com/article/10.3390/healthcare12070807/s1. File S1: PRISMA Checklist. Table S1: Search strategies. Table S2: The methodological quality assessment of the included studies. Table S3. Individual factors related to outbreaks. Table S4. Individual factors related to COVID-19-related deaths. Table S5. Organizational factors related to infection outbreaks. Table S6. Organizational factors related to COVID-related deaths. Table S7. Environmental factors related to COVID outbreaks. Table S8. Environmental factors related to COVID-related deaths. References [127,128] are cited in the supplementary materials.
