# Supplementary material for: Drivers of COVID-19 Outcomes in Long-Term Care Facilities Using Multi-Level Analysis: A Systematic Review
Source: Healthcare (Basel). 2024 Apr 8;12(7):807. doi: 10.3390/healthcare12070807 (PMC11011537; doi:10.3390/healthcare12070807)
Supplement: Supplementary file 1 [file healthcare-12-00807-s001.zip › Table S1. Search strategies..pdf]

Table S1. Search strategies.

Searches were conducted in OVID MEDLINE, EMBASE, EBSCOhost CINAHL, and Wiley Cochrane Database of Systematic Reviews. The search results were limited to those published since 2022 and in English.

Below are the numbers of results retrieved from each database:

- MEDLINE: 499
- EMBASE: 636
- CINAHL: 163
- Cochrane Database of Systematic Reviews: 45

There are 961 results after deduplication.

Below are the search strategies.

Ovid MEDLINE(R) ALL 1946 to May 22, 2023

#	Searches	Results
1	exp Coronavirus/ or Coronavirus Infections/ or COVID-19/ or (covid or coronaviru* or corona viru* or ncov* or n-cov* or novel cov* or COVID-19 or COVID19 or COVID-2019 or COVID2019 or SARS-CoV-2 or SARSCoV-2 or SARSCoV2 or SARSCoV19 or SARS-Cov-19 or SARSCov-19 or SARSCoV2019 or SARS-Cov-2019 or SARSCov-2019 or severe acute respiratory syndrome coronaviru* or severe acute respiratory syndrome cov 2 or 2019 ncov or 2019ncov).kf,tw.	377723
2	Long-Term Care/ or residential facilities/ or assisted living facilities/ or homes for the aged/ or nursing homes/ or intermediate care facilities/ or skilled nursing facilities/	77885
3	(long term care or assisted living or continuing care or extended care or ((residential or nursing or senior* or old age or geriatric* or elder*) adj (lodge* or facilit* or home* or residence*))).kf,tw.	68605
4	2 or 3	106494
5	disease outbreaks/ or disease hotspot/	91085
6	mortality/ or "cause of death"/ or fatal outcome/ or hospital mortality/ or survival rate/	386626
7	(outbreak* or case rate* or positiv* rate*).kf,tw.	174212
8	(mortalit* or death rate* or fatality rate* or "cause* of death" or death cause* or fatal outcome* or survival rate* or survival time*).kf,tw.	1278682
9	or/5-8	1667006
10	1 and 4 and 9	1260
11	limit 10 to (english language and yr="2022 -Current")	499

EBSCOhost CINAL			
#	Query	Limiters/Expanders	Results
S1	((MH "COVID-19") OR (MH "SARS-CoV-2")) OR TI (covid or coronavirus* or corona viru* or coronavirinae* or covid2019 or covid19 or covid-19 or nCoV* or n-CoV* or novel CoV* or 2019-nCoV* or 2019nCoV or 19nCov or hCoV* or h-Cov* or 2019-hCoV* or 2019hCoV* or 19 hCoV* or SARS-CoV-2 or SARSCoV2 or SARSCov-2 or SARS-CoV-19 or SARSCoV19 or SARSCoV-19 or SARS-Cov-2019 or SARSCoV2019 or SARSCoV-2019 or "severe acute respiratory syndrome CoV 2" or "severe acute respiratory syndrome coronavirus 2") OR AB (covid or coronavirus* or corona viru* or coronavirinae* or covid2019 or covid19 or covid-19 or nCoV* or n-CoV* or novel CoV* or 2019-nCoV* or 2019nCoV or 19nCov or hCoV* or h-Cov* or 2019-hCoV* or 2019hCoV* or 19 hCoV* or SARS-CoV-2 or SARSCoV2 or SARSCov-2 or SARS-CoV-19 or SARSCoV19 or SARSCoV-19 or SARS-Cov-2019 or SARSCoV2019 or SARSCoV-2019 or "severe acute respiratory syndrome CoV 2" or "severe acute respiratory syndrome coronavirus 2")	Expanders - Apply equivalent subjects<br>Search modes - Boolean/Phrase	127,876
S2	(MH "Long Term Care") OR (MH "Residential Facilities") OR (MH "Nursing Homes+") OR (MH "Skilled Nursing Facilities") OR (MH "Assisted Living")	Expanders - Apply equivalent subjects<br>Search modes - Boolean/Phrase	58,093
S3	TI (long term care or assisted living or continuing care or extended care or ((residential or nursing or senior* or old age or geriatric* or elder*) N (lodge* or facilit* or home* or residence*))) OR AB (long term care or assisted living or continuing care or extended care or ((residential or nursing or senior* or old age or geriatric* or elder*) N (lodge* or facilit* or home* or residence*)))	Expanders - Apply equivalent subjects<br>Search modes - Boolean/Phrase	23,704
S4	S2 OR S3	Expanders - Apply equivalent subjects<br>Search modes - Boolean/Phrase	67,818

S5	(MH "Disease Outbreaks") OR (MH "Disease Hotspot")	Expanders - Apply equivalent subjects<br>Search modes - Boolean/Phrase	41,873
S6	TI (outbreak* or case rate* or positiv* rate*) OR AB (outbreak* or case rate* or positiv* rate*)	Expanders - Apply equivalent subjects<br>Search modes - Boolean/Phrase	32,300
S7	(MH "Cause of Death") OR (MH "Hospital Mortality") OR (MH "Mortality")	Expanders - Apply equivalent subjects<br>Search modes - Boolean/Phrase	66,706
S8	TI (mortalit* or death rate* or fatality rate* or "cause* of death" or death cause* or fatal outcome* or survival rate* or survival time*) OR AB (mortalit* or death rate* or fatality rate* or "cause* of death" or death cause* or fatal outcome* or survival rate* or survival time*)	Expanders - Apply equivalent subjects<br>Search modes - Boolean/Phrase	293,258
S9	S5 OR S6 OR S7 OR S8	Expanders - Apply equivalent subjects<br>Search modes - Boolean/Phrase	372,132
S10	S1 AND S4 AND S9	Expanders - Apply equivalent subjects<br>Search modes - Boolean/Phrase	593
S11	S1 AND S4 AND S9	Limiters - Published Date: 20220101-20231231; English Language<br>Expanders - Apply equivalent subjects<br>Search modes - Boolean/Phrase	163

Embase 1974 to 2023 Week 20

#	Searches	Results
1	COVID-19/ or SARS-CoV-2/ or coronavirinae/ or betacoronavirus/ or Coronavirus infection/ or (covid or coronavirus* or corona viru* or coronavirinae* or covid2019 or covid19 or covid-19 or nCoV* or n-CoV* or novel CoV* or 2019-nCoV* or 2019nCoV or 19nCov or hCoV* or h-Cov* or 2019-hCoV* or 2019hCoV* or 19 hCoV* or SARS-CoV-2 or SARSCoV2 or SARSCov-2 or SARS-CoV-19 or SARSCoV19 or SARSCoV-19 or SARS-Cov-2019 or SARSCoV2019 or SARSCoV-2019 or "severe acute respiratory syndrome CoV 2" or "severe acute respiratory syndrome coronavirus 2").kw,tw.	458595
2	long term care/ or assisted living facility/ or nursing home/ or residential home/ or home for the aged/	213113

3	(long term care or assisted living or continuing care or extended care or ((residential or nursing or senior* or old age or geriatric* or elder*) adj (lodge* or facilit* or home* or residence*))).kf,tw.	89692
4	2 or 3	240761
5	disease hotspot/	452
6	(outbreak* or case rate* or positiv* rate*).kf,tw.	206917
7	mortality/ or all cause mortality/ or death toll/ or excess mortality/ or hospital mortality/ or mortality rate/	1097946
8	(mortalit* or death rate* or fatality rate* or "cause* of death" or death cause* or fatal outcome* or survival rate* or survival time*).kf,tw.	1897126
9	or/5-8	2351775
10	1 and 4 and 9	2231
11	limit 10 to (english language and yr="2022 -Current")	771
12	limit 11 to medline	135
13	11 not 12	636

Wiley Cochrane Database of Systematic Reviews

ID	Search	Results
#1	[mh "coronavirus"] or [mh ^"coronavirus infections"] or [mh "COVID-10"] or (covid or ositive us* or corona viru* or ncov* or n-cov* or novel cov* or COVID-19 or COVID19 or COVID-2019 or COVID2019 or SARS-CoV-2 or SARSCoV-2 or SARSCoV2 or SARSCoV19 or SARS-Cov-19 or SARSCov-19 or SARSCoV2019 or SARS-Cov-2019 or SARSCov-2019 or severe acute respiratory syndrome ositive us* or severe acute respiratory syndrome cov 2 or 2019 ncov or 2019ncov):ti,ab,kw	1523181
#2	[mh ^"long-term care"] or [mh ^"residential facilities"] or [mh ^"assisted living facilities"] or [mh ^"homes for the aged"] or [mh "nursing homes"]	2220
#3	(long term care or assisted living or continuing care or extended care or ((residential or nursing or senior* or old age or geriatric* or elder*) Next (lodge* or ositive* or home* or residence*))):ti,ab,kw	38911
#4	#2 or #3	39153
#5	[mh ^"disease outbreaks"] or [mh ^"disease hotspot"]	280
#6	(outbreak* or case rate* or ositive* rate*):ti,ab,kw	25351

#7	[mh ^"mortality"] or [mh "cause of death"] or [mh "fatal outcome"] or [mh "hospital mortality"] or [mh "survival rate"]	20885
#8	(mortalit* or death rate* or fatality rate* or "cause* of death" or death cause* or fatal outcome* or survival rate* or survival time*):ti,ab,kw	197445
#9	#5 or #6 or #7 or #8	215531
#10	#1 and #4 and #9	8400
#11	Limit #8 to Cochrane Reviews	473
#12	Limit #9 to Publication Date: 01/01/2022 to 05/23/2023	45
