# Supplementary material for: Drivers of COVID-19 Outcomes in Long-Term Care Facilities Using Multi-Level Analysis: A Systematic Review
Source: Healthcare (Basel). 2024 Apr 8;12(7):807. doi: 10.3390/healthcare12070807 (PMC11011537; doi:10.3390/healthcare12070807)
Supplement: Supplementary file 1 [file healthcare-12-00807-s001.zip › Table S2. The methodological quality assessment of the included studies..pdf]

Table S2. The methodological quality assessment of the included studies.

First Author	Year	QAS* Level	First Author	Year	QAS* Level	First Author	Year	QAS* Level
**Rabilloud**	2023	2	Gorges	2021	1	Panagiotou	2021	2
**Abrams**	2020	3	Harrington	2020	2	Peckeu-Abboud	2022	2
**Adler**	2022	2	He	2020	2	Piet	2021	2
**Agoües**	2021	1	Hege	2021	2	Preus	2022	2
**Aguilar-Palacio**	2022	2	Heras	2021	2	Rabilloud	2022	2
**Akhtar**	2021	2	Hill	2022	3	Rutten	2020	1
**Arendse**	2022	2	Houben	2023	2	Sacco	2020	2
**Arienti**	2021	3	Hua	2022	1	San Roman	2023	2
**Arnedo-Pena**	2022	1	Ibrahim	2021	2	Scanferla	2023	1
**Bagchi**	2021	3	Iyanda	2022	2	Shallcross	2021	1
**Booij**	2022	2	Kain	2021	2	Shen	2022	2
**Brown**	2021	2	Kennelly	2021	2	Shi	2020	2
**Bui**	2020	2	Khairat	2021	2	Soldevila	2021	2
**Cai**	2021	2	Kim	2022	2	Stall	2020	2
**Cangiano**	2020	2	Kumar	2021	2	Sugg,	2021	1
**Cazzoletti**	2021	2	Lane	2022	1	Suñer	2021	2
**Chatterjee**	2020	3	Lee	2021	2	Tang	2020	2
**Chen**	2021	2	Lee	2022	2	Tarteret	2021	2
**Chen**	2020	1	Levy-Storms	2022	2	Telleria	2022	1
**Cooper**	2021	1	Li	2022	1	Thorsness	2022	2
**Corvol**	2022	2	Li,Temkin-Greener, Cen, et al.	2020	1	Torres	2022	2
**Cox**	2023	1	Li, Temkin-Greener, Shan, et al.	2020	2	Travers	2021	2
**Cronin**	2022	1	Longo	2022	2	Unruh	2020	2
**Dean**	2020	2	Lord	2021	2	Veronese	2021	2
**Emmerson**	2022	2	Lu	2021	2	Vijh	2021	2
**Engeda**	2023	2	Mas Romero	2020	2	Visser	2023	2
**Escriba**	2022	1	Mattingly	2021	2	Weech-Maldonado	2021	1
**FigueroA**	2020	2	McGarry	2021	1	Williams	2021	2
**Fisman**	2020	2	Mehta	2021	2	Young	2023	2
**Frigotto**	2023	2	Meis-Pinheiro	2021	2	Zhang	2022	1
**Gilman**	2021	3	Morciano	2021	2	Zhu	2022	1
**Gopal**	2021	2	Olson	2022	2	Zimmerman	2021	2
**Gorges**	2020	1	Orlando	2022	2	Zunzunegui	2022	2

*** QAS=** Quality assessment scores per the Joanna Briggs Institute's critical appraisal tools ranked the studies into three levels: High quality (Level 1): Studies that meet most or all of the criteria and demonstrates minimal potential for bias. Moderate quality (Level 2): Studies that meet some of the criteria or exhibits a moderate likelihood of bias. Low quality (Level 3): Studies that do not meet multiple criteria or exhibit a significant risk of bias.
