# Supplementary material for: Drivers of COVID-19 Outcomes in Long-Term Care Facilities Using Multi-Level Analysis: A Systematic Review
Source: Healthcare (Basel). 2024 Apr 8;12(7):807. doi: 10.3390/healthcare12070807 (PMC11011537; doi:10.3390/healthcare12070807)
Supplement: Supplementary file 1 [file healthcare-12-00807-s001.zip › Table S3. Individual factors related to outbreaks..pdf]

Table S3. Individual factors related to outbreaks.	
**Socio-demographic background**	**Age:** Older, rather than younger age [24,46,60,63,97,101,105,115] was associated with outbreaks.<br>In one 500-bed long-term care facility, the outbreak was found to be more prevalent among younger older adult individuals in an unadjusted analysis model [105].<br>In a study of an LTCF with predominantly frail older residents with significant neurocognitive disorders, a 100% likelihood of COVID-19 [Relative Risk (RR): 17, 95% Confidence Interval (CI): 2.5-113.8] was documented for:<br>- residents younger than 90 (no age range provided) with dyspnea and falls,<br>- residents older than 90 (no age range provided) with anorexia and<br>- residents older than 90 (no age range provided) without anorexia but with altered consciousness [39].
	**Sex:** Female was associated with a higher risk of infection and represented 75% of the COVID-19 population [45,56]. Examining shifts throughout 2020 and the initial months of 2021, a female sex (Relative Risk: 1.16, Confidence Interval: 1.07–1.25) was noted as a determinant for occurrences in LTCFs during the later phase of the pandemic (December-February 2021) [91].<br>A significant association between male sex and outbreaks in a large academic LTCF [105] as well as with substantial outbreaks (positive cases >10% of licensed beds) but in an unadjusted analysis ($p = 0.03$) [99].
	Concerning **socio-economic status**, the percentage of families below poverty level (RR: 1.15, CI: 1.05–1.26) was a predictor of COVID-19 cases in the mid-pandemic (September-December – 2020) period [91]
**Condition-specific factors**	**Comorbid conditions**, including respiratory disease [60], peripheral vascular disease, hypertension, anemia [107], bowel incontinence [105], urinary incontinence [58], chronic kidney disease, asthma, and cancer [107] were associated with outbreaks. Diabetes was identified as having an association with COVID-19 outbreaks in certain studies [105,107]. However, in another study, this association was on the brink of statistical significance, with a p-value of 0.06 [116]. Those with stroke had 4.10 times the risk of symptomatic COVID-19 than those without stroke [57,105]. Residents with behavioural symptoms and cognitive deterioration/dementia also had a higher probability of infection [105,107]. In a study of 673 LTCFs, the odds of the positive test in LTCFs in which 50–74% of residents had dementia were 3.36 times compared to those with non-dementia residents. The odds increased to 4.54 times in facilities with more than 75% of residents with dementia[115].<br>In a study of 15 LTCF, depression exhibited a connection with the outbreak and continued to show a notable correlation with a heightened risk of infection, even after adjusting for factors like age, gender, and the specific facility [107]. However, another study carried out across four LTCFs observed that among participants diagnosed with dementia and having a GDS (Global Deterioration Scale) score of ≥ 6, those who accounted for the sample were found to be 35% less susceptible (adjusted odds ratio [aOR]: 0.65, $p < 0.037$) to SARS-CoV-2 infection. The researchers postulated that the substantial presence of residents with dementia (68%) could have influenced these findings. [116].
	**Smokers** were more likely to be infected by COVID-19 (*p*-value=0.008) [107].

	**Co-existent medications**, including antidementia drugs (AChEI/Memantine) and antipsychotic medications, were more likely to be taken by those without COVID-19 symptoms [58]. It was felt unlikely that these medications protected against viral infection, and rather that residents with dementia are pauci-symptomatic, and frequently report few, if any, symptoms [58]
	**Seroprevalence level:** LTCFs where individuals exhibited intermediate seroprevalence (with 50%-70% of individuals testing seropositive for IgG response against the SARSCoV-2 nucleoprotein), as well as LTCFs with high seroprevalence (where more than 70% of individuals were seropositive), experienced reductions of 55% and 78% respectively in the risk of outbreaks when compared to LTCFs with low seroprevalence individuals[61].
	Increasing **BMI** was associated with about 6% higher risk of infection [101], while several other studies explored it with no significant associations.
	**Resident dependency**, as measured by the Barthel scores [46,54] and Tinetti (Performance Oriented Mobility) scores [46], showed an association between increased dependency and a greater risk for infection [46].<br>The need for more care and having greater staff contacts (e.g., residents with severe cognitive or functional impairment or with a feeding tube, intravenous line, or catheter), higher medical pre-existing condition, a higher level of needs, problems with mobility, and disability in basic activities of daily living (BADL) were associated with a higher risk of infection [22,58,63,72,101,105].
	A higher **Frailty Index** (greater degree of frailty) was associated with outbreaks among residents [24] and COVID-19 symptoms manifestation [58]. Almost a 30% increase in the odds of a positive test for severely frail residents (1.29 95% CIs 1.05–1.59) was reported compared to mildly, moderately or not frail individuals [115].
	Higher **instruments score**, including the Resident Assessment Instrument – Minimum Data Set (RAI-MDS), Changes in Health, End-stage disease and Signs and Symptoms (CHESS) was more likely to be associated with outbreaks [24].
	An estimated poor **life expectancy** was associated with a greater than 6% increase in the risk of infection [101].
	Lower **duration of stay (<100 days) in LTCFs** was found to be significant for the likelihood of large outbreaks (number of cases > 10% of licensed beds) even after adjustment analysis [99].
	**Hospitalization** in general [58] and the number of residents previously hospitalized /weekly average number of residents [113] were also associated with outbreaks.
