# Supplementary material for: Drivers of COVID-19 Outcomes in Long-Term Care Facilities Using Multi-Level Analysis: A Systematic Review
Source: Healthcare (Basel). 2024 Apr 8;12(7):807. doi: 10.3390/healthcare12070807 (PMC11011537; doi:10.3390/healthcare12070807)
Supplement: Supplementary file 1 [file healthcare-12-00807-s001.zip › Table S4. Individual factors related to COVID-19 related deaths..pdf]

**Table S4. Individual factors related to COVID-19 related deaths.**	
**Socio-demographic background**	Greater **age** was significantly associated with death within 30 days after a positive COVID-19 test [24,53,59,101,103,115].<br>Mortality was significantly higher among the older & oldest-old, ("oldest old" defined as those aged 80 or over and while "older" is commonly used to describe individuals in the age range of 65 to 80.) [24,29,33,46,51,58,60,75,98];and residents who survived COVID-19 were more likely to be of younger age [46]. The higher risk of mortality was significantly higher among those ≥ 80 years old compared to 65–79 years old at all 7, 30, and 90 days[55].<br>The odds ratio of death for those aged 90 years or older was 2.14-2.94 compared to younger residents, falling within the range of 65 to 79 years.[101,103]. A one-year increase in the average age of residents was associated with a 0.3% higher COVID-19 mortality percentage (P < 0.01)[102], and every 10-year increase in age increased the risk of death by 1.33 fold (95% CI 1.23– 1.45)[32], Older age groups of residents with COVID-19 had significantly higher odds of 30-day all-cause mortality compared with those less than 65 years old [the older, the higher odds) [68]. One study reported 22.7% of COVID-19 cases died, of which 24.8% were COVID-19-related deaths and found the adjusted incidence rate for older age was significantly 4% higher than younger for all-cause resident mortality[66].
	**Male sex** was at higher risk [59,62,101,103] and was significantly linked with COVID-19-related deaths compared to females [20,24,29,32,46,51,52,54,58,60,98,103]. A significantly higher hazard rate for death within 30 days for men ranged from 58- 69% in two studies [24,68]while being female was significantly associated with a lower risk of mortality within 7, 30, and 90 days after COVID-19 diagnosis[55]. In an adjusted hazard ratio model, a significantly higher probability (32%) was also evident for men than women (aHR, 1.32; 95% CI, 1.29-1.35); [101].
	A higher score on the **social engagement level** was a predictor of lower mortality risk [29].
**Condition-specific factors**	**Cognitive deterioration** was linked to an elevated likelihood of COVID-19-related fatalities, with odds ranging from 1.28 to 1.68 and hazard ratios ranging from 1.34 to 1.45[20,29,32,33,59,98,101] at 90 days [55,115]. Individuals with Alzheimer's disease and related dementias had 33.4 excess deaths per 100,000 in comparison to those without these conditions [86].
	Higher **comorbidity/ Charlson Comorbidity Index** in general [24,52,55,58,98]and longer duration of co-existent comorbidities [29] were associated with death. High complexity was associated with a higher risk of death within 30 days after COVID-19 diagnosis[55].<br>Chronic disease, including heart failure/cardiovascular disease [32,55,60,68,101], hypertension [60,81,110] , end-stage disease [24], diabetes [101,103,107], chronic kidney disease [68,101,103,107] at 90 days [55], ESRD [98], reduced kidney function [51], hepatopathy [59], complex chronic condition [60,62], respiratory disease [60,101], pneumonia [45,59], respiratory symptoms [20], immunocompromised status, inflammatory and immune factors and lower lymphocyte count [20,29,98], prior hospital contacts for respiratory illness [29], obesity [110], Parkinson's disease

	[51], bowel incontinence [58], and auditory impairment [58], depression [101], GDS ≥ 6 (had almost five times higher probability) [116] hallucinations/ aggressive behavior [101], history of COPD and emphysema [107] (with seven times more probability) [116] were all associated with a higher number of deaths than not having those conditions; except for cancer, for which divergent findings were identified[68,101], and for obesity within 90 days after COVID-19 diagnosis[55]
	Residents with multiple **symptoms** generally had the highest mortality risk [107].<br>Symptoms linked to mortality were fever [20,58,59,103], fatigue [49], confusion [58], hypoxia/ respiratory insufficiency [49,58,103], dyspnea and stupor and refusal of oral intake [59], pneumonia [45,59],and tachycardia [103],
	**Laboratory tests** identifying lower hemoglobin concentration, lymphocyte count, serum albumin, and more elevated potassium and glomerular filtration rate (GFR) were all associated with an increased likelihood of mortality [29]. Kidney function as an independent predictor of COVID-19 mortality was studied using an estimated glomerular filtration rate (eGFR) and identified the risk of death ranged from 1.4 times higher for the group with an eGFR 60-89 to 2.3 times higher for the group with an eGFR <15 compared with the group with an eGFR >90 [108]. A lower mortality risk was also associated with a higher total HDL cholesterol ratio, hemoglobin, lymphocyte count, glomerular filtration rate, platelet count and total iron binding capacity [29].<br>The higher mortality risk is more likely for higher iron saturation and urate [29].
	Poor **nutrition** (high risk 7.6%, med risk 8.6%) and **dehydration** (high risk 2.4%, low risk 2.4%)were associated with a higher risk of death [29].
	While a high **BMI** was associated with a high risk of infection, mortality was significantly increased among those with a low BMI [46,101]compared with a BMI of 18.5 to 25 [101].<br>Conversely, survivors of COVID-19 were more likely to have normal BMI [46].
	Increased **dependency levels** and worse activities of daily living (ADL) scores [2] detected from instruments such as the Barthel index [20,46,54,116], Tinetti scale [46], Scheda di Osservazione Intermedia dell'Assistenza" (SOSIA) frailty classification [46], or acuity index among minorities residents [110]were associated with increased mortality [44,54,103,110,113].<br>Conversely, residents who survived COVID-19 were more likely to have better ADL scores [46].<br>With the increasing **level of care needed**, functional impairment [32,101] or severe disability [56], the chance of death statistically significantly increased [63,113].
	The presence of **frailty** (defined based on the items used in the Multidimensional Prognostic Index [MPI], the Frailty, and Frail-VIG index) and Pre-frail condition were significantly related to a greater hazard and odd ratio [24,116]and a statistically significantly increased probability of COVID-19-related death [44,46,105,115,116]. The proportion of death significantly increased along with the increase in the frailty severity (16.7% in prefrail, 22.2% in moderately frail, and 50.0% in frail) [105]. Females with high frailty levels (SOSIA) accounted for 73% of all the deaths [45]. Residents who died from COVID-19 had lower handgrip strength but not statistically significant [23].
	**Co-existent medications:**

	Collectively, the total number of medications was significantly associated with 30-day COVID-related mortality[52].<br>The impact of medications individually was reported as follows:<br>***Hydroxychloroquine therapy*** was associated with a reduced likelihood of death [46], but was subsequently refuted [127].<br>***Hydroxychloroquine and azithromycin*** were associated with a higher likelihood of survival [20].<br>***Statin*** intake was associated with an increased likelihood of death [58]. ***Vitamin D*** supplementation was associated with a reduced likelihood of death [46].<br>***Antipsychotic use*** were significantly associated with threefold greater odds[116].<br>***Benzodiazepines*** was associated with 2.23 times higher odds but an unadjusted mode [116].<br>***Hyperpolypharmacy*** (≥10 medications) was associated with 2.25 times higher odds but an unadjusted model [116].<br>***Proton pump inhibitor*** was associated with increased mortality (p=0.004) [52].<br>***Antiplatelet therapy*** was associated with a decrease in the rate of death (p=0.026) [52].<br>***Anticoagulation*** lowered the risk of mortality [46] by 30% (RR 0.70 95% CI 0.51–0.96) [32]. The use of antiplatelets and anticoagulants was significantly associated with lower odds (17%) of 30-day mortality compared to those not using anticoagulants (aOR 0.83, 95% CI 0.71-0.97) [68].
	A more prolonged **duration of stay in LTCFs** was a predictor for a lower mortality risk [29].
	A COVID-19-related **hospitalization** was associated with a statistically significantly greater risk of death [58]. The number of residents previously admitted or readmitted to the hospital for COVID-19 treatment per week [113], hospitalization for acute myocardial infarction [98] or for respiratory infection since 2000 and the number of ED visits with a respiratory diagnosis in the prior year [29] were associated with risk of death. Palliative medical management was also associated with higher risk of mortality at 6 months [49].
	**COVID-19 rates** in LTCFs were significantly associated with higher mortality among residents in LTCFs[66].
