# Supplementary material for: Drivers of COVID-19 Outcomes in Long-Term Care Facilities Using Multi-Level Analysis: A Systematic Review
Source: Healthcare (Basel). 2024 Apr 8;12(7):807. doi: 10.3390/healthcare12070807 (PMC11011537; doi:10.3390/healthcare12070807)
Supplement: Supplementary file 1 [file healthcare-12-00807-s001.zip › Table S5. Organizational factors related to infection outbreaks..pdf]

Table S5. Organizational factors related to infection outbreaks.	
**LTCFs quality indicators**	Seven studies identified a significant inverse association between the **star rating** (quality ranking) of LTCFs and COVID-19 cases [59,63,69,83,88,89,111]. The higher rating was associated with a lesser spread of resident infections following an initial case [74] and lower COVID-19 incidence [111], and was found to have less (1/3) COVID-19 cases than low-star rating facilities [88]. More specifically, four- or five-star LTCFs had 13% fewer COVID-19 cases than one- to three-star facilities [94]. In contrast, a positive association between the quality star ratings and a large outbreak was found (p = 0.04) but it became insignificant when analysis adjusted for outbreaks in counties, licensed beds, lower CNA staffing hours, and proportion of short-stay residents [99]. Similarly another study found this positive association but only the higher star-rated LTCF located in communities with a low proportion of minorities [70].<br>Star ratings were also analyzed in terms of their subset domains:<br>- ***Concerning nursing staffing***: three studies identified a significant association between high ratings (based on the mean staffing hours per resident) and lower COVID-19 cases [76,79,82,113].<br>- ***Concerning quality measures and health inspection domains:*** three studies identified an inverse association between the level of star rating and COVID-19 cases[79,99,113]. A higher probability [71] and odds [82] of having COVID-19 residents were reported in LTCFs with lower star ratings and higher total health deficiencies, defined as violations of federal regulations [71,82,106].
	**Quality performance** was also captured in nine other quality indicators domains:<br>- ***Substantiated complaints*** [71] and the ***number of fines*** [106]; both were related to increased risk of COVID-19 transmission in LTCFs [106] and a higher number of COVID-19 cases [71].<br>- LTCFs with ***emergency preparedness deficiencies*** had a higher mean number of COVID-19 cases than those without [71].<br>- A higher number of ***unmet items in a standardized assessment tool*** at the onset of COVID-19 exposure or outbreak was associated with larger outbreaks [31].<br>- LTCFs which reported ***difficulties in isolating*** residents had significantly higher odds of outbreaks and large outbreaks compared with LTCFs that did not [34].<br>- LTCFs who ***implemented outbreak management procedures*** were more likely to have no cases of COVID-19 than those with at least one case of COVID-19 [43].<br>- ***Frequently employing agency nurses or caregivers*** were associated with increased odds of infection in residents, outbreaks, and large outbreaks compared with those that did not [34].
**Staffing**	**Staffing levels** [76,79,80,82,106,113] and staffing shortages demonstrated a high degree of interconnectedness [28], a statistically significant association with outbreaks [34,82,84,106], a reduced odds of infection in residents [34]. More specifically:<br>- Each unit increase in the ***staff-to-bed ratio*** was found to be statistically significantly associated with reduced odds of infection in residents [34].

	LTCFs with more COVID-19 cases were statistically significantly more likely to have fewer direct care hours per resident per day [72] and total nursing hours[26]. A relative increase in the risk of 41% incidence proportion was found for a higher residents/staff ratio (aRR = 1.41 95% CI: 1.09–1.82)[56].<br>- The ***total RN hours*** were inversely related to COVID-19 infection rates [59,82,97]. The higher proportion of RNs was associated with a 0.626% lower infection rate [48].<br>- The odds of outbreaks in LTCFs that had low RN hours (less than 0.75 hours per resident day [hprd]) were two times higher than those that did not [82].<br>- LTCFs with fewer cases were more likely to have a higher RN staffing level (.8 hprd with zero cases and .6 hprf for LTCFs with 1–10 and 11–69 cases) [94].<br>- The probability of a significant reduction in COVID-19 cases by an increase in RN hours (1 hprd) in LTCFs located in high-minority communities was three times higher than in those in low-minority communities [70,82].<br>- ***CNA hours*** were inversely associated with COVID-19 infection rates [26,80,97,99]. The incidence proportion was 38% higher for a higher residents/nursing assistant ratio (aRR = 1.38 95% CI: 1.24–1.54) [56] and was 11% higher with a higher beds/nursing assistant ratio (aRR = 1.11 95% 1.01–1.22)[56]. Higher COVID-19 case rates was also significantly associated with the lower state-level CNAs' training requirements[92].<br>Across three stages of the pandemic in 2020 and early 2021, the higher overall staffing was a predictor for experiencing lower COVID-19 cases during the early- and mid-pandemic but not during the late pandemic (December 2020 -February 2021). More specifically, the rate was 28% lower during the early-pandemic (May- September – 2020) (RR 0.72 CI 0.61–0.84 P<0.001) while it was 13% lower during mid-pandemic (September-December – 2020) (RR 0.87 CI 0.80–0.93 P<0.001) [91].<br>Inconsistent results were reported for the association of staffing levels/ number of direct care employees on outbreak status [41,69–72,80,89,91,98,100,106].
	The presence of **infected staff** was associated with the **COVID-19** outbreak [36,42,54,89,113] and was linked to a five- to 11-fold [36] COVID-19 cases and almost 50 times (aRR = 49.7 95% CI: 33.0–78.2) increase in incidence proportion [56]. Another study also referred to an infected staff member as a strong predictor with which the risk of an outbreak became 154.7 % [47].
	Concerning **the assignment of nursing staff to residents** in LTCFs, where staff provided care for all residents, regardless of COVID-19 status, the odds of infection cases and outbreaks were significantly higher than those where staff were segregated and only cared for infected or uninfected residents separately [22,34].
	Regarding **employment status**, An alpha variant outbreak among residents was reported 4.49 times more likely in LTCFs with staff employed in a maximum of 2 facilities than in LTCFs with staff employed in a maximum of one ward. (aOR: 4.49, 95% CI: 1.11-18.07, p = 0.035) [50]. Subcontracted professional nursing and subcontracted nonregulated services were significantly associated with the outbreak[26].
	In LTCFs with ***labour unions***, COVID-19 cases were statistically significantly less likely to occur than those without unions [75,102].

	Statutory sick pay to staff was significantly associated with reduced odds of infection and large outbreaks[34,75].
**Ownership and membership affiliation**	Concerning **type of ownership**, government-owned LTC or non-profit facilities [63] and locally owned, state-owned LTCFs and non-profit agencies [84] had significantly lower infection rates than for-profit LTCF [63,84].<br>The COVID-19 positivity percentage in privately owned and non-profit Long-Term Care Facilities (LTCFs) exhibited a 3-3.64 fold and 1.8-fold significant increase, respectively, when compared to municipally owned LTCFs [24,57]. Likewise, another study demonstrated an adjusted hazard ratio signifying a notably higher risk of outbreaks in non-profit LTCFs in comparison to facilities owned by health authorities [26]. The likelihood of a significantly elevated outbreak risk was calculated to be 99% for for-profit LTCFs when contrasted with health authority–owned facilities [26].<br>Study of the outbreaks in different time intervals and the first two waves (March 1 to July 31 and August 1 to December 31, 2020) in France showed the reduction of the hazard of outbreak by 44% in public hospital-associated LTCFs compared to in private for-profit ones (hazard ratio 0.56, 95% CI 0.40-0.80 P=.001). The significant reduction was also reported for public non-hospital-associated, private not-for-profit, and public hospital-associated LTCFs compared to private for-profit ones during the 2nd wave (August 1-December 31, 2020). 44%44[37].<br>For-profit status was associated with the following:<br>- a higher proportion of LTCFs with COVID-19 residents [82], a higher number of COVID-19 cases [72,113].<br>- a 1.96-12.7 times higher in the outbreak size than non-for-profit [6,30,79],<br>- a significantly higher odds of infection in residents [30,34],<br>- large outbreaks [30], which defined as a third or >20 cases of staff and residents combined being infected [30,34], and<br>- a 16.7% higher incidence of infection [97].<br>In contrast, one study found the risk of infection in public (government-owned) LTCFs was significantly higher than for-profit [64].<br>In the total effects of variables in the structural equation, for-profit facilities, larger LTCFs and more infected staff had greater numbers of infected residents. For-profit facilities were more likely to have lower quality ratings (average rating of 2.5 vs average rating for non profit 4.2), larger size, and a lower ratio of staff, which increased the number of infected staff [89].
	Non-**chain membership status** was significantly ($p< 0.05$) related to an increased probability of having a COVID-19 case [67], while in four studies, chain membership was found to be associated with a higher likelihood of having COVID-19 cases [30,70,97] 6% [97] in the LTCFs specifically those in a high-minority community [70,97].<br>**Privately owned by large chain** LTCFs were more likely to have an outbreak [21].
**Medicare and Medicaid coverage**	Facilities with higher **Medicaid-insured residents** (mean= 59.3%) reported higher cases compared to those that did not (Mean= 56.7%)[71] and were found statistically significantly associated with outbreaks [72,73,97]. More specifically, LTCFs with high numbers of Medicaid residents had 16% more COVID-19 cases than those who did not [94].

	A separate study found that among the nursing homes included in the study sample, those with higher proportions of racial/ethnic minority residents, which were associated with increased odds of cases, were also more likely to have a larger number of Medicaid-covered residents [95].The sources of payment as a predictor of COVID-19 cases in LTCFs differed across three stages (early, mid, late) of the pandemic in the southeastern part of the U.S. While the proportion of LTCF residents whose payment source was **Medicare** was a predictor of COVID-19 cases in the mid-pandemic (September-December – 2020) period, in the early period, proportion of residents whose payment source was **Medicaid** was the predictor of COVID-19 cases in LTCFs [91].
**LTCF's racial and ethnic composition**	LTCFs serving a high proportion (defined as greater than 16.26% - 50%) of minorities were associated with a higher outbreak probability [67,70,73,77,81,83,84,95,99,105,106,109]; specifically, these facilities experienced a 15% more confirmed cases compared to those with a lower proportion of minority residents [94].<br>After adjusting for various factors including nursing home, county, and state variables, a study found that compared to nursing homes with low proportions of racial/ethnic minority residents (<2.92%), the likelihoods of experiencing at least one new resident case increased by 25% for medium proportions (2.92%–11.11%), 44% for medium-high proportions (11.11%–30.18%), and 76% for high proportions (≥30.18%) of racial/ethnic minority residents. The statistical significance (P-values) associated with these increases were 0.025, 0.002, and <0.001, respectively [95]. More specifically, the adjusted risk ratio for Black residents, Hispanic residents, and Asian residents was 1.56, 1.72, and 1.63 respectively[101].<br>Another study showed LTCFs with any Black residents were statistically significantly more likely to have COVID-19 infections than those without and suggested this finding probably reflects the structural bias that may underlie resources, services, and provider inequities [109].<br>Examination of the total effects of variables in the structural equation showed that LTCFs with a higher number of White residents were in neighbourhoods with lower levels of social vulnerability, less likely to be for-profit facilities and, were associated with higher quality ratings [89].
