# Supplementary material for: Drivers of COVID-19 Outcomes in Long-Term Care Facilities Using Multi-Level Analysis: A Systematic Review
Source: Healthcare (Basel). 2024 Apr 8;12(7):807. doi: 10.3390/healthcare12070807 (PMC11011537; doi:10.3390/healthcare12070807)
Supplement: Supplementary file 1 [file healthcare-12-00807-s001.zip › Table S6. Organizational factors related to COVID-related deaths..pdf]

Table S6. Organizational factors related to COVID-related deaths.	
**LTCFs quality indicators**	An inverse association between the **star rating** of the LTCF and the mortality rate was reported[63,81,88,111]. The death rate was 30% [88] and 24.2% [74] higher among 1-star compared to 5-star LTCFs. The highest death counts by racial composition were also reported among 1-star LTCFs with ≤ 59.7% White residents [81].<br>Concerning the star rating subdomains, including ***staff rating, quality measures rating, and health inspection domains,*** an inverse association between the domain rating and the death rate was reported [98,113]. Fully-adjusted mixed model in a study showed that COVID-19 deaths were higher in the LTCFs in the lowest quartile of facility ratings and least in facilities with high facility ratings (including quality measure rating, health inspection rating, staff rating, short-term rating, longtime rating, and registered nurse staff rating) [87].
	**Quality performance** was also reported concerning six other relevant quality indicators:<br>- ***Unpreparedness for a pandemic*** was associated with a higher likelihood of death. More specifically, for each one-point increase in the 12-point score of unmet measures, the mortality risk increased by 5% [62].<br>- ***Hospital-dependent LTCFs*** were more likely to have a decreased death rate than non-hospital-dependent LTCFs [40].<br>- ***LTCFs with union workers*** had a 30 percent relative decrease in death rate compared with LTCFs without unions [75].<br>- ***A daily resident clinical examination, monitoring vital signs*** three times per day and ***providing prophylactic anticoagulation*** during the first three weeks of the outbreak was associated with a lower mortality rate than in LTCFs not using these practices [40].<br>- Poor decision-making in ***initiating a clinical protocol for urinary incontinence*** was associated with a higher risk for mortality [29].
**Staffing**	Concerning **staffing levels,** total nursing hours were associated with a lower mortality rate [80]. The decrease in death rate was 0.088% when there was a higher proportion of RNs [48].<br>Conversely, fewer hours of nursing care [81] was associated with an increased risk of COVID-19 mortality. High-minority LTCFs, which had higher rate of mortality had significant nursing and other staffing shortages [110].<br>Every 20-minute increase in ***RN staffing*** [94] and more direct care hours in LTCFs with ≥100 Beds [2] was statistically significantly associated with fewer COVID-19 deaths.<br>A higher ratio of ***CNA staffing*** to residents was statistically significantly associated with lower COVID-19 mortality rates [75,80,92,98]. Inversely, the mortality rate is 44% more likely to increase with a higher resident/nursing aid ratio[56]. Higher COVID-19 mortality was associated with lower state-level CNAs' training requirements[92].<br>However, higher registered nurse (RN), Licensed Practical Nurse (LPN) [98] and certified nursing assistant (CNA) hours in high-ethnic minority communities [70] were associated with a slight increase in severe COVID-19 outcomes [98] and a statistically significantly higher probability of death [41,70].<br>Higher staff size, including those not involved in care, was associated with worse outcomes and suggested reducing the number of unique staff (RN, LPN, CNA) members

	and maintaining direct care hours by considering full-time instead of part-time staff [100].
	The number of **infected staff** was associated with the number of resident deaths [27,66,89,113]. The mortality rate was almost 60 times more likely among residents with COVID-19 (59.90 95% CI: 26.64–134.89)[56].
**Ownership and chain affiliation**	**For-profit and/or private LTCFs,** compared to government/state-owned nursing facilities and non-profit, municipal homes, were more likely to have COVID-related deaths [2,24,30,56,63,83,87,98,113]. For-profit LTCFs are associated with an additional .53 deaths per 100 beds [104]. For-profit ownership was associated with 1.3% higher mortality percentage ($P < 0.05$) [102].<br>In private LTC homes, the likelihood of death was 25% higher than in municipal homes [24].<br>However, a higher death rate was also more likely in not-for-profit LTCFs [21]. One study also found the risk of death in public (government-owned) LTCFs was significantly higher than in for-profit[64].
	Other types of ownership, such as **chain membership**, were associated with a higher likelihood of death [70,75,104] in high-minority communities [70,75,104]. More specifically, they were significantly associated with an additional .35 deaths per 100 beds [104]. Chain organization affiliation was associated with 0.7% higher mortality percentage ($P < 0.05$)[102].<br>LTCFs belonging to a corporate chain/ groups of providers (branded) had higher odds of COVID-19-related death than non-branded LTCFs [33].
**Medicaid/ Medicare coverage**	**Receipt of Medicaid coverage** was associated with an 8.6 percentage point greater probability of death [2,81]. A 1% higher enrollment in the **Medicare** program was associated with 0.06% lower mortality percentage ($P < 0.01$)[102].
**LTCF's racial and ethnic composition**	The relationship between a LTCF's **racial and ethnic composition** and death outcomes was inconsistent:<br>A statistically significantly higher probability of death was found among LTCFs with a high proportion of minorities (i.e., Blacks and Hispanics) compared to LTCFs with a low proportion [2,67,70,81,83,85,95,109,110]. A study of the trends in racial and ethnic disparities in the early periods of the pandemic (April 13- June 19, 2020) showed a 26% relative increase in the difference in the fatality rate ratio over time compared to counterpart facilities [96].<br>Reverse associations were also obtained [63,75,103]; LTCFs with a minority of White residents reported a lower number of COVID-19 deaths than LTCFs with a majority of White residents. This result became insignificant by including region-level fixed effects [75].<br>Examination of the total effects of variables in the structural equation showed that LTCFs with a higher number of White residents had neighbourhoods with lower levels of social vulnerability and were less likely to be for-profit and, thus, were associated with higher quality ratings and had fewer resident COVID-19 death [89].<br>Inconsistencies emerged in the outcomes of studies examining three parameters.

	- The comparison **among different states** within the USA regarding the relationship between LTCFs serving predominantly white patients and the likelihood of decreased COVID-19 mortality rate [2].<br>- **The temporal trend was observed during the pandemic**[78,90]; during the mid-2020 period, mortality rates were elevated in LTCFs catering to minority populations, while in the latter months of the year, the rates were higher among LTCFs with predominantly white residents.<br>- Focusing on **minorities within minorities**, a significant number of deaths were reported in LTCFs in the USA that housed a considerable proportion of Black and Hispanic residents [70,81]; however, two studies indicated an association with higher mortality rates related to Asian race/ethnicity.[98,101]. The adjusted risk ratio for Asian ethnic composition was highest (1.25), while the probability was lower, 1.02 for Black and 1.04 for Hispanic or Latino compared to White race [101].
