# Supplementary material for: Drivers of COVID-19 Outcomes in Long-Term Care Facilities Using Multi-Level Analysis: A Systematic Review
Source: Healthcare (Basel). 2024 Apr 8;12(7):807. doi: 10.3390/healthcare12070807 (PMC11011537; doi:10.3390/healthcare12070807)
Supplement: Supplementary file 1 [file healthcare-12-00807-s001.zip › Table S7. Environmental factors related to COVID outbreaks..pdf]

Table S7. Environmental factors related to COVID outbreaks.	
**Community Factors**	**The outbreaks in counties/communities** were significantly associated with outbreaks in local LTCFs [26,29,31,60,69,79,80,84,93,104], size of outbreak [38,91,104] and with the odds of a COVID-19 outbreak in LTCFs [30].<br>The incidence of COVID-19 among residents and staff members in LTCFs was similar to the surrounding community [6].<br>In one study, this association was linked to a large outbreak (defined as the number of residents with COVID-19 > total of 10% of licensed beds) even after adjustment [99]. A significant association was also identified for the increase in the incidence of hospitalization of the community population for COVID-19[37].
	**High-density communities** (higher population density) /population square miles, average household size [106], large metropolitan, urban, and large community size were associated with increased COVID-19 cases in LTCFs [21,29,56,67,73,106].<br>However, divergent results were identified over time; the outbreak rate was influenced significantly by the location population density during the second wave (August 1 to December 31, 2020) but not in the first wave ((March 1 to July 31)[37].
	**Staff from a community with a high rate of COVID-19** and public transportation use [104] were more likely to be associated with the outbreak in LTCFs and remained significant in the adjusted model[105].
	Concerning **the location of LTCF,** the geographic area was associated with the risk of infections [24,43], where a COVID-19 outbreak was more likely in those located close to a high-risk industry (e.g., abattoirs) than in LTCFs not in those settings [21,56].
	**Community sociodemographic status** was associated with LTCF outbreaks in two ways:<br>- **The racial/ethnic composition of the community** in which LTCFs were located was associated with LTCF outbreaks; while minority populations were linked to reduced cases among residents in LTCFs [63], the outcomes for LTCFs resident cases showed variability based on the diverse racial compositions within the community. For instance, a higher percentage of non-Hispanic Asians in the community was linked to decreased LTCFs resident cases. Conversely, a higher percentage of the Hispanic population was associated with lower levels of NH resident cases [113], A study investigated the correlation between county racial and ethnic composition and COVID-19 infection rates, examining changes over time. Initially, in the early stages of the pandemic (June and before), nursing home infection rates disproportionately affected racial and ethnic minority populations. However, as time progressed, counties with elevated percentages of Black, Asian, and Hispanic populations observed a gradual decline in these rates, commencing from mid-July onwards. In contrast, counties with higher proportions of individuals from various racial backgrounds, such as multiple races, American Indian/Alaska Native, and Native Hawaiian/Other Pacific Islander, witnessed a progressive increase in nursing home-associated COVID-19 infection rates, reaching a peak between November and December 2020 [84]. The interaction between LTCFs' racial/ethnic composition and the county's/communities' characteristics for the COVID-19 outbreak was reported [63,70], including a

	significant association between the racial and ethnic composition of LTCFs and their communities and the likelihood of having COVID-19 cases [70].<br>- Concerning **socio-economic status**, an inverse association between the extent of outbreaks in LTCFs and the income status of the neighbourhoods [24,77,84,97,106]. and high median annual personal income in the county was reported. More specifically, the risk of COVID-19 cases was 44% and 28% higher in the LTCFs of neighbourhoods of low and medium socioeconomic position, respectively, compared with those in neighbourhoods of high position[64]. Other studies also found that low-income levels and a higher percentage of minorities in surrounding counties were both associated with increased risks of infection in LTCFs [24,84,106]. Unemployment in the surrounding area was associated with an increased risk of COVID-19 transmission in LTCFs [106].
**LTCF Physical characteristics**	A higher **number of beds** (in general or on the basis of ≥100 beds or >50 beds) was significantly linked with outbreaks [21,26,31,54,56,60,63,65,67,73,82,83]. A study reported 5.37 times more likely to have an outbreak in facilities with more than 15 beds than those with fewer than 15 beds. Another study found a 75%, 58%, and 61% higher rate of COVID-19 cases when it comes to higher certified beds during May- September 2020 (RR: 1.75, CI: 1.64–1.88), September-December, 2020 (RR: 1.58, CI: 1.48– 1.70), and (December-February, 2021) (RR: 1.61, CI: 1.48–1.74) respectively [91].<br>Considering the impact of capacity during different COVID-19 waves in France, during the first wave(March 1 to July 31 2020), the probability of outbreak increased significantly with the LTCF capacity (P < .001). However, during the second wave (August 1 to December 31, 2020), this association was for a capacity of nearly 80 beds (P <.001) [37]. The threshold for the extent of capacity varied in two other studies as the larger LTCFs with greater than 140 beds reported a twofold higher percentage of positive cases compared to smaller LTCFs (<100 beds, [6,99].<br>Considering the impact of capacity across different COVID-19 variants, Alpha variant outbreaks, not the classic variant outbreaks, had a strong association (aOR: 8.10, 95% CI: 2.50-26.24, p <0.001) with the large ward size (≥21 beds) compared with small ward size (≤10 beds)[50].
	A high **crowding index** [25], **occupancy rate** [30,83,97] **resident census** [84,94,106] **and** an increase in the number of **new admissions**[34]in an unadjusted analysis [47]were significantly associated with increased odds of infection in residents and outbreaks. The risk of outbreaks was 1.91 times greater in medium LTCFs (50–100 residents) and 4.57 times greater in large LTCFs (>100 residents) compared to small LTCFs (less than 50 residents) [61]. The incidence proportion was twice more likely to increase with a raised crowding index[56]. The cumulative incidence rate of residents was two times the risk with a raised occupancy rate [56]. However, in one study, LTCFs with more COVID-19 cases had lower occupancy rates [72].)
	Concerning the **structural design of the rooms** in LTCFs, the higher number of single/ private rooms, certified beds (which was used as a proxy for facility size) and a larger living area per bed were inversely associated with the likelihood of an outbreak [21,25,104,113].

	A small outbreak (≤10 cases per 100 residents) was more likely to occur where there were single rooms for each resident (70.8%) [21].<br>A larger outbreak was significantly associated with shared rooms [21,25,113]. Similar results were identified but in an unjustified model[26].
	**Older design standards of the LTCFs** were associated with the odds of a COVID-19 outbreak (adjusted OR 1.55, 95% CI 1.01–2.38) [30].<br>**Facility age** was associated with COVID-19 cases [83], and the incidence proportion increase was 6% in higher-age LTCF buildings [56]. The outbreak rates were six times greater in LTCFs opened before 1972 than those opened after 1972 (adjusted RR 5.9, 95% CI 2.3-14.9) [31].
	In the LTCFs employing **the greenhouse model (**which serves 10 to 12 residents, with consistent staff assignment, private rooms and bathrooms, a smaller overall space, and a central entry), the incidence of COVID cases per 100 residents was half that of small LTCFs with 50 beds [114]. Those in small LTCFs were 2.68 times more likely to be infected than those in green houses (IRR=2.68; 95% CI: 2.03, 3.54)[112].
	The presence of **ventilator-dependent units** significantly decreased the chance of higher residents' total COVID-19 outbreaks [63,113] for each week of access [113].
	The **mechanical recirculation of air** significantly increased the odds of the classic variant outbreak 4.43 more likely than those without (aOR 4.43 95% CI: 1.26- 15.61 P 0.02) [50].
	Concerning the **type of care provided in the ward**, the odds of Alpha variant outbreaks in wards providing psychogeriatric care was 3.11 more likely (aOR: 3.11, 95% CI: 1.04-9.33, p = 0.043) than those providing somatic care[50].
