# Supplementary material for: Drivers of COVID-19 Outcomes in Long-Term Care Facilities Using Multi-Level Analysis: A Systematic Review
Source: Healthcare (Basel). 2024 Apr 8;12(7):807. doi: 10.3390/healthcare12070807 (PMC11011537; doi:10.3390/healthcare12070807)
Supplement: Supplementary file 1 [file healthcare-12-00807-s001.zip › Table S8. Environmental factors related to COVID-related deaths..pdf]

**Table S8. Environmental factors related to COVID-related deaths.**	
**Community Factors**	In general, higher **community or county** incidences of outbreaks were associated with an increase in COVID-19-related LTCF deaths [60,80,104,113] among residents in racially and ethnically mixed LTCFs[77].<br>In addition, the hospitalization for COVID-19 in the community was significantly associated with the cumulative number of deaths in LTCFs. It was three times higher during the second wave (August 1 to December 31, 2020) than the first wave (March 1to July 31, 2020) [37].
	**High-density communities** (higher population density) was significantly associated with the cumulative number of death in LTCFs during both waves (March 1 to July 31 and August 1 to December 31, 2020) in France [37]
	Higher rates of **staff public transportation use** were associated with an additional 1.4 deaths per 100 beds [104].
	A lower risk of death was found for those receiving **disability support programs** [29].
	Concerning **the location of LTCF,** significant differences in excess mortality in geographic region was found [2,24,87]. For instance, in contrast to LTCFs in Connecticut and New Jersey, a significantly lower probability of 6 or more deaths in LTCFs in New York with high percentages of White residents with COVID-19 was found [2]. Facilities located in rural areas [62] and near a high-risk industry [21] had higher mortality rates than homes without those characteristics. There is a higher death prevalence in LTCFs in large fringe metropolitan areas than in non-core or less-urbanized areas [87].
	**Community sociodemographic status** was also associated with LTCF mortality in two ways:<br>- In communities with high **proportions of ethnic minorities,** the probability of LTCFs COVID-19 death was inconsistent as indicated by studies [63,70]. In alignment with this, another study revealed that diverse racial groups in community settings have differing effects on LTCFs resident deaths. Specifically, a higher percentage of non-Hispanic Asians in the community is associated with lower LTCFs resident deaths [113]. Between the county's and LTCF's racial/ethnic demographic composition and its association with COVID-19 deaths, there were incompatible interactions [63,70]. However, it was observed that the high-minority LTCFs in low-minority communities had an overall decreasing trend in the likelihood of COVID-19 deaths but had an increasing trend in high-minority communities [63,70]. The proportion of non-Hispanic Asians in the county was inversely associated with the LTCF's COVID-related death rate [113].<br>- LTCFs being located in more **socially deprived communities** were significantly associated with an increase in the rate of COVID-19-related mortality [104]. Considering various neighbourhood socioeconomic levels, a study found that the risk of death was 51% and 26% higher in the LTCFs in neighbourhoods with low and medium socioeconomic positions, respectively, compared to those with high socioeconomic positions [64].
**LTCF Physical Characteristics**	LTCFs with fewer than 100 **beds** were associated with reduced COVID-related death rates [2,62,63], while in counties with COVID-19 incidence beyond 250/100,000 COVID-19, mortality sharply increased with LTCFs size [65]. A significantly higher odds of COVID-19-related death were reported by the large LTCFs [41+ beds] and the

	medium-sized LTCFs [24-40 beds] than small-sized LTCFs [33]. In another study, the risk of death in medium-sized LTCFs was reported higher by 2.73 times (95% CI 1.23–6.07) compared to smaller-sized facilities [32]. Very small and large LTCFs were found in another study to have higher COVID-19 mortality, whereas LTCFs with 30 to 70 places had the lowest level, [65]. However, one study observed that a greater number of beds, as a place with more resources, was associated with a lower risk of mortality [29]. Similarly, death risk was found lower for LTCFs with over approximately 100 residents [98]. More specifically, an increase of ten occupied beds was associated with a 0.1% lower mortality percentage ($P < 0.1$) [102].
	LTCFs with high **levels of crowding [104]** had a twice higher mortality incidence, and high crowding was associated with an increased relative risk of mortality compared to low-crowding LTCFs. The incidence of proportion was three times more likely to increase with a raised crowding index[56]. The occupancy rate was also significantly associated with increased death rates [2,25,56,64,83,104]. One study reported LTCFs with more deaths were more likely to be large with a high resident census [94]. Conversely, another study observed death risk increased with increasing number of residents in smaller LTCFs [98].
	Concerning the **structural design of the rooms** in LTCFs, a higher number of single/private rooms[66], more certified beds, and a greater area per bed, led to statistically significantly fewer deaths [21,113]. Living in multiple-bed vs. single-bed rooms and 4-bed rooms versus 2-bed rooms increased the mortality rate by 30.1% and 18.1%, respectively [25].
	**Facility age** was associated with mortality, and the rate increase was 8% in higher-age LTCF buildings [56]
	Using the **greenhouse model** of LTCF care provision was associated with a decrease in mortality compared to even small LTCFs (with fewer than 50 beds) [112,114], and it was discussed that while residents of greenhouse facilities are older and sicker, the COVID-19 related death is less likely. Those in small LTCFs were 2.54 times more likely to die than those in small LTCFs (IRR= 2.54; 95% CI: 1.33–4.82)[112].
	For each week increase in the number of weeks with a **ventilator-dependent unit,** the incidence of resident death was decreased by 1.2 % [113].
